# Modelling of human torso shape variation inferred by geometric morphometrics

**DOI:** 10.1371/journal.pone.0265255

**Published:** 2022-03-10

**Authors:** Michael Thelwell, Alice Bullas, Andreas Kühnapfel, John Hart, Peter Ahnert, Jon Wheat, Markus Loeffler, Markus Scholz, Simon Choppin

**Affiliations:** 1 Advanced Wellbeing Research Centre, Health Research Institute, Sheffield Hallam University, Sheffield, United Kingdom; 2 LIFE Research Center for Civilisation Diseases, Leipzig University, Leipzig, Germany; 3 Institute for Medical Informatics, Statistics and Epidemiology, Leipzig University, Leipzig, Germany; 4 IFB Adiposity Diseases, Leipzig University, Leipzig, Germany; The Cyprus Institute, CYPRUS

## Abstract

Traditional body measurement techniques are commonly used to assess physical health; however, these approaches do not fully represent the complex shape of the human body. Three-dimensional (3D) imaging systems capture rich point cloud data that provides a representation of the surface of 3D objects and have been shown to be a potential anthropometric tool for use within health applications. Previous studies utilising 3D imaging have only assessed body shape based on combinations and relative proportions of traditional body measures, such as lengths, widths and girths. Geometric morphometrics (GM) is an established framework used for the statistical analysis of biological shape variation. These methods quantify biological shape variation after the effects of non-shape variation–location, rotation and scale–have been mathematically held constant, otherwise known as the Procrustes paradigm. The aim of this study was to determine whether shape measures, identified using geometric morphometrics, can provide additional information about the complexity of human morphology and underlying mass distribution compared to traditional body measures. Scale-invariant features of torso shape were extracted from 3D imaging data of 9,209 participants form the LIFE-Adult study. Partial least squares regression (PLSR) models were created to determine the extent to which variations in human torso shape are explained by existing techniques. The results of this investigation suggest that linear combinations of body measures can explain 49.92% and 47.46% of the total variation in male and female body shape features, respectively. However, there are also significant amounts of variation in human morphology which cannot be identified by current methods. These results indicate that Geometric morphometric methods can identify measures of human body shape which provide complementary information about the human body. The aim of future studies will be to investigate the utility of these measures in clinical epidemiology and the assessment of health risk.

## Introduction

Anthropometry refers to the scientific procedures and processes of acquiring surface anatomical measures of the human body–height, weight, lengths, and girths–by means of specialist equipment [1]. Medical practitioners measure the outside of the human body to assess various aspects of an individual’s health, guide their treatment and evaluate prevalence of disease within the population [2–4]. Large-scale anthropometric surveys have been fundamental to epidemiological studies of the distribution and determinants of health-related states or events in specified populations during the last century [3, 4]. In practice, manual anthropometrics are the dominant method used to assess physical health during initial screenings, as well as population-level diagnosis of disease risk and obesity prevalence [5, 6]. Height and weight are the most common measures [7, 8], and are typically combined to calculate an individual’s body-mass-index (BMI: kg/m^2^). BMI provides a measure of relative obesity and stratifies individuals into categories (underweight, normal weight, overweight and obese) according to World Health Organization (WHO) guidelines [9, 10]. However, simply using BMI to assess physical health should be performed with caution. BMI is unable to identify several health risk factors, such as the ratio of fat to lean tissue [11] and distributions of fat around the body [4, 12], which have been shown to have greater implications for adverse metabolic effects [4, 13].

Waist girth is also commonly used in health assessments, due to its ease of capture, as well as stronger relationships with visceral adiposity volume [14], lean mass [15] and morbidity risk [16–18] compared with BMI. It has also been suggested that the torso is the segment of the human body that has the greatest potential for differences in size and shape, due to individual variations in fat and lean tissue present within the abdomen [19, 20]. Practitioners often calculate ratios of different torso girth measures to assess central adiposity and abdominal shape, such as the waist-hip ratio (WHR), waist-chest ratio (WCR), waist-height (WHtR) or waist-by-height^0.5^ ratios (WHT.5R) [14, 17, 21–23]. These have also been shown to have greater associations with quantities of abdominal visceral fat and health risk, compared to BMI [21, 24]. WHO suggests combining one of these central obesity measures with BMI to improve the identification and treatment of at-risk individuals [4, 10]. However, this approach for assessing body shape is limited and does not fully describe the complex variations in human form [25–28]. More sophisticated, scale-invariant measures are required to identify subtle curvatures and contours present on the external human body [17, 29, 30] and improve the characterisation of relationships between human morphology and health risk [25].

Three-dimensional (3D) imaging systems capture detailed and accurate point cloud data that provides a representation of the surface of a 3D object. This point cloud data contains the differential geometric properties of the objects surface, from which both size and shape characteristics can be extracted. 3D imaging is an established anthropometric tool within several applications, including apparel sizing [31–33], and for clinical evaluation and health monitoring [28]. Due to the rapid and repeatable acquisition of 3D imaging data, previous studies have collected body measures from large participant samples [17, 25, 34], and used machine learning to establish clusters of individuals that exhibit similar traits based on traditional measures [25, 34]. However, it is increasingly recognised that creating shape variables by creating linear combinations of one-dimensional traditional body measures is a relatively simple approach which discards the shape information captured by 3D imaging systems [28]. More recently, principal components analysis (PCA) [30, 35] and surface curvature [36, 37] have been used to assess variations in human body shape and estimate its underlying composition.

Geometric morphometrics (GM)—methods for analysing variations in the shape of biological organisms [38]—quantify biological shape variation using the coordinates of anatomical landmarks after the effects of non-shape variation (location, rotation and scale) have been mathematically held constant. Torres-Tamayo et al. [39, 40] previously used GM to investigate thoracic-pelvic covariation in the skeletal structures of humans, showing that their morphological variation is heavily influenced by sexual dimorphisms associated with different bioenergetic demands between males and females. Shape-based measures of the human torso have also been shown to complement current anthropometrics in the estimation of torso fat quantities [41]. However, it is yet unclear whether shape measures identified using geometric morphometrics provide additional information compared to traditional body measures. If variations in human torso shape cannot be fully explained by current anthropometric techniques or sex-related morphological differences between males and females, this would suggest that additional measures could be beneficial in evaluating aspects of human morphology that could improve health risk assessments in practice.

The aim of this study was to determine whether shape measures, identified using geometric morphometrics, can provide information that cannot be obtained using current techniques. The objectives were to determine the inter-dependence between traditional body measures and features of torso shape within a large sample and identify variations in torso shape which are unexplained by current anthropometric techniques.

## Materials and methods

### Study and participants

In this study 3D imaging data collected in the Leipzig Research Center for Civilization Diseases LIFE-Adult study was analysed. LIFE-Adult is a population-based cohort study, which collected phenotype data of over 10,000 randomly sampled individuals from the city of Leipzig, Germany, covering a main age range from 40–79 years of age, with a subset of 400 participants aged 18–39 years. Comprehensive details of the study design can be found in Loeffler et al. [2]. As a prerequisite to enrollment, written informed consent was obtained from all participants. The study protocol adheres to the principles laid out in the Declaration of Helsinki. The LIFE-Adult study was approved by the ethics board of the Medical Faculty of Leipzig University. All procedures and documents for this study were approved by the Sheffield Hallam University Research Ethics Committee, reference number ER13534279.

Basic characteristics of participants considered in the present analysis are shown in Table 1 (full details can be found in S1 Table). We performed a cross-sectional analysis to model variations in human morphology identified using geometric morphometrics. The ‘Strengthening the Reporting of Observational Studies in Epidemiology’ (STROBE) statement for cross-sectional studies was followed in the development of this manuscript [42].

**Table 1:** Summary characteristics of LIFE-Adult participants considered in the present analysis.

	Male			Female		
Descriptive	Mean (SD)	Min.	Max.	Mean (SD)	Min.	Max.
No. of participants	4,405			4,804		
Age (years)	58 (13)	18	81	57 (12)	19	80
Height (cm)	176.1 (7.3)	150.5	206.5	164.0 (7.0)	141.6	188.4
Weight (kg)	86.0 (14.5)	49.6	174.7	72.1 (14.3)	40.0	182.8
BMI (kg/m^2^)	27.6 (4.2)	16.5	50.2	27.0 (5.3)	16.2	55.6
WHR (cm/cm)	0.99 (0.07)	0.74	1.21	0.87 (0.07)	0.65	1.16

### Body measurement protocol

3D imaging data of participants was acquired using a commercial Vitus Smart XXL 3D laser scanner (Human Solutions GmbH, Kaiserslautern, Germany), in accordance with ISO 20685–1:2018, international standards for 3D scanning methodologies for internationally compatible anthropometric databases [43]. Details of the full experimental protocol are described in [2]. For each participant, 140 body measures were automatically extracted from each 3D image using Anthroscan ScanWorX software (version 2.9.9.b, Human Solutions GmbH) based on the location of anatomical landmarks, determined by proprietary automatic landmark identification algorithms. See S1 Table for a full list of extracted body measures. We showed in a previous study that automatically extracted body measures are in good agreement with their classical anthropometric counterparts (if available) [44].

### Data analysis

#### Normalisation of body measures

All extracted body measures related to body segments outside of the torso region of interest (e.g., ankle height or upper arm length) were removed from the analysis. The resulting data matrix consisted of 34 body measures for each participant (see S1 Table). To facilitate comparisons between individuals of differing heights, the torso body measures of each participant were normalised by body height. Finally, height normalised torso body measures were converted into z-scores, providing a common scale in units of standard deviations from the mean.

#### 3D imaging data post-processing

**Fig 1:**
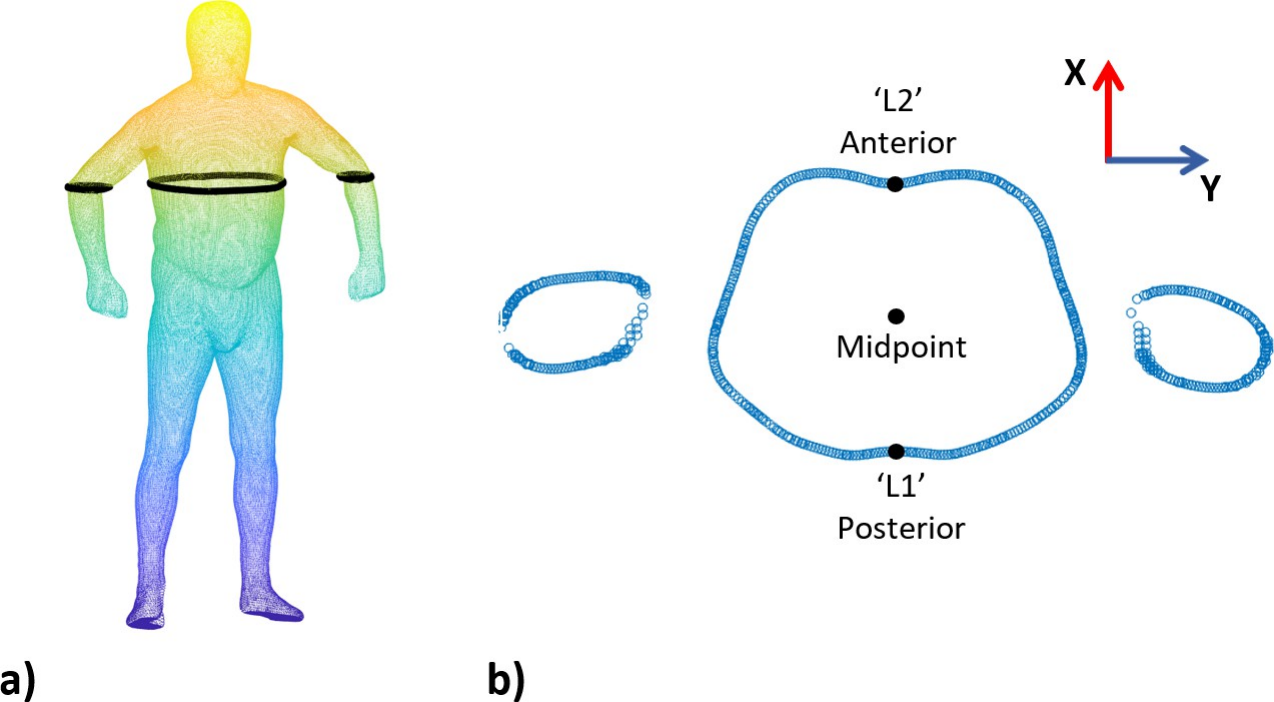
Identification of anterior and posterior torso landmarks. (a) Extracted data points from 3D image, (b) Identification of anterior and posterior landmarks within point slice data.

A modified version of the lower trunk segment proposed by Wicke [20] was used to define the upper and lower limits of the torso segment, as the area encompassed between the xiphoid process and the buttock landmark, respectively (Fig 1). The buttock landmark corresponded to the gluteal (hip) girth location, as defined by the International Society for the Advancement of Kinanthropometry (ISAK) [45] (p84). The height of the xiphoid process was chosen as the superior boundary of the torso segment to prevent complications in scan segmentation caused by occlusion in the axilla (armpit) region, as in previous studies [41, 46]. In a small investigation with a sample of 100 individuals, the vertical height of the xiphoid process was found to be 60% ± 1.5% of the distance between the buttock and neck height landmarks, as detailed in a previous study by the authors [47].

The following procedure was used to determine the circumferential location of the xiphoid process landmark on the anterior torso aspect and a landmark corresponding to the spinous process on the posterior aspect. To identify the circumferential location of the xiphoid process and spinous process a band of data points was extracted from each 3D image at the height of xiphoid process (Fig 1A). The local maxima and minima of the extracted data points along the x-axis were identified on the posterior and anterior aspects of the torso respectively (Fig 1B). These localised peaks were assumed to correspond to each location, which would otherwise be manually palpated.

**Fig 2:**
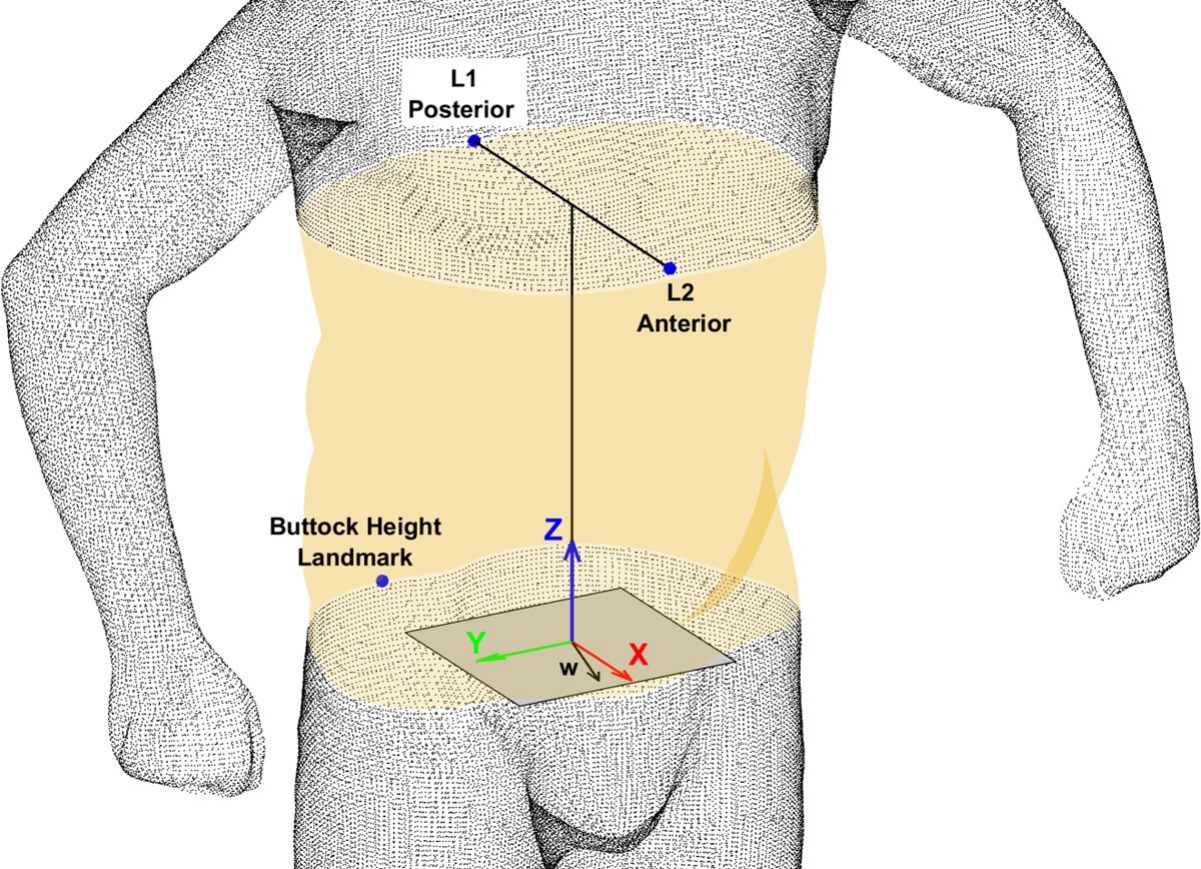
Landmarks used to segment the torso and create a local co-ordinate system within each 3D image.

To remove non-shape variation (scale, location, orientation) from the data, an anatomical coordinate axis system was created at the centre of each torso segment, according to the convention defined in ISO 20685–1:2018 [43]. The x-axis was aligned in the sagittal direction, the y-axis was aligned in the transverse direction and the z-axis was aligned longitudinally (Fig 2). The centre of the torso was defined as the midpoint between the posterior (L1) and anterior (L2) landmarks identified on the surface of the 3D image. The x-axis (X) was initially defined as the vector $\overset{⃑}{L1\;L2}$. The z-axis (Z) was defined as the cross product of X and a second vector on the x-y plane (w), which was defined as the vector X rotated by 15° about the Z-axis of the global co-ordinate system. The y-axis (Y) was defined as the cross product of X and Z: *Y* = *X*×*Z*.

Following alignment, each 3D image was constrained to only include the coordinate points relating to the torso segment region of interest (Fig 2). Twenty-five separate, 2 mm bands of 3D coordinate data points were extracted from each torso at uniform intervals along its length. The height of each band of data points was set at 2 mm to ensure that the external shape features of the torso segments were preserved while allowing for any gaps in the point cloud, based on a previous study by Clarkson et al. [48]. The z-coordinate of each raw data point within each extracted band of points was set to the same value, corresponding to the position of that band within the torso segment, thereby creating individual 2D outline shape profiles along the length of the torso. Finally, these shape profiles were rescaled according to centroid size to remove the effects of scale between participants in the sample. Centroid size is the most commonly used size metric for this scaling operation within geometric morphometrics, and is defined as the square root of the sum of squared distances of landmarks from the centroid of a configuration matrix of points [38]. The centroid size (CS) of a configuration of points (*X*) is denoted mathematically as:

$$CS(X)=\sqrt{\sum_{i=1}^{K}\sum_{j=1}^{M}{\left(X_{ij}-C_{j}\right)}^{2}}$$

Where K is the number of landmarks, M is the number of coordinates and *C_j_* is the location of the *j*th component of the centroid. The centroid size of all extracted shape profiles along the length of each torso was scaled by a single scale factor, making the sum of distances from each point to the centroid for all slices along the torso segment equal for all participants. This removed any differences in scale between participants, enabling the analysis of torso shape according to statistical shape theory.

#### Torso shape feature detection

Following alignment, cubic smoothing splines [49] were fit to each extracted shape profile. A Fast-Fourier transform (FFT) algorithm was used to transform each shape profile into the frequency domain. Coefficients above 10 were determined to be noise within the signal waveform (by assessing the RMSE difference between the original and filtered signal) and were removed. As a result, the number of FFT coefficients used to describe each shape profile was reduced from 35 to 10, meaning that 250 coefficients were needed to describe the torso segment of each participant.

Principal component analysis (PCA) was used to further reduce the dimensionality of the torso segment’s 3D data. Previous studies have demonstrated that body shape variations are subtle, requiring a greater number of principal components (PCs) to describe them fully [30, 35, 41]. It was decided that PCs which accounted for less than 1% of total variance in the dataset would be omitted from subsequent analysis. This procedure produced a feature vector that characterises the torso shape of each participant. All 3D imaging data processing and feature detection procedures were performed using MATLAB (MathWorks, Natick, USA). The MATLAB code used to process the raw 3D imaging data and detect the torso shape PCs are available in S1 File.

### Statistical analysis

Histograms and Q-Q plots were visually inspected, and a Shapiro-Wilks test was conducted to assess the normality of all extracted body measures and shape features within SPSS (IBM SPSS Statistics 24.0). Independent t-tests were conducted to determine if there were statistically significant differences between male and female participants in all 34 body measures and shape PCs (see S1 Table). In addition, to explore whether possible sex-related differences in torso shape are a consequence of differences in body measures between females and males, a preliminary partial least squares (PLS) regression analysis between body measures and individual shape PCs of all participants was conducted. The resulting regression residuals of each shape PC (dependent variable) were then regressed on sex (independent variable) in a binary form (females = −1, males = 1) (see S1 Table). If this regression showed a significant result, this would suggest that the unexplained shape variations within that PC, could result from sexual dimorphisms between males and females. However, if this regression was not significant, this would suggest that the unexplained shape variations within that PC are not sex-related but could in fact be the result of some other explanatory factor, such as mass distribution or associated health risk. Pearson’s product-moment correlations, *r*, were used to assess associations between individual body measures, as well as between body measures and shape PCs. P-values < 0.001 were considered statistically significant, to account for the effect of large sample sizes (as opposed to the typical alpha level of 0.05 [50]. The coefficient of determination, *R*^2^, was calculated as a measure of effect size, assessing the practical importance of correlations between individual variables independent of sample size [50]. Correlations and effect sizes were calculated using SPSS (IBM SPSS Statistics 24.0).

PLS regression models were used to determine how the torso shape PCs related to the 34 body measures. Since torso shape PCs were unrelated, separate PLS regression models (with traditional anthropometrics used as predictor variables) were created for each one shape PC, following recommendations by Wold [51, 52]. Ten-fold cross-validation was performed to determine the number of components required for each PLS regression to avoid overfitting, which was (assessed using mean squared prediction error, MSPE). To determine the importance of each body measure within each PLS regression, the variable importance in projection (VIP) statistic was calculated. VIP is the weighted sum of squares of the PLS-weights (the amount of Y-variance of each PLS component [52, 53]. Wold [52] suggests that predictor variables with a VIP ≥ 0.8 can be considered to significantly contribute to the model and have high predictive power. PLSR, MSPE and VIP calculations were performed using MATLAB (Mathworks, Natick, USA).

Sex-specific models were created so that trends between traditional body measures and shape PCs could be compared for each sex. Separate PLS regression models were created to estimate changes in each torso shape PC from unit changes in waist girth. Changes in torso shape were estimated across the entire range of waist girth measures within the sample, with height measures held constant at 180 cm and 160 cm for the male and female models, respectively.

### Availability of data

Pre-processed torso shape PCs of all participants analysed in this study, together with their age, sex, BMI and WHR are provided in S2 Table. In this work we only analysed point cloud data of the torso segment. However, it cannot be ruled out that raw data can be assigned by image comparison and thus we cannot consider torso segment point cloud data as anonymised. Since raw data was accessed via a data sharing agreement with the administration of the LIFE study at Leipzig University, interested readers must contact them for raw data access. Raw 3D data can be requested from the LIFE research centre (www.life.uni-leipzig.de/en/).

## Results

### Torso shape features

**Fig 3:**
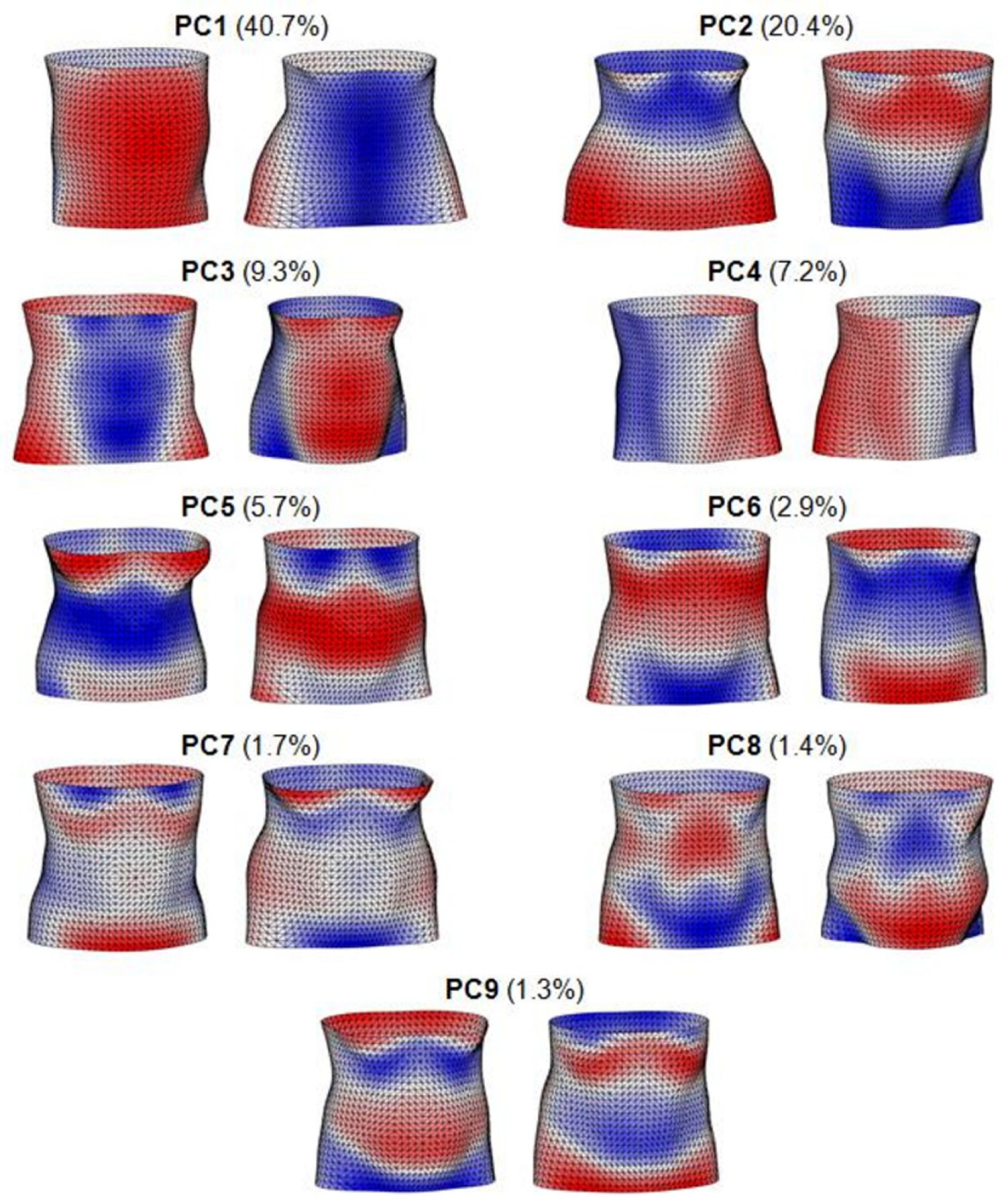
Torso shape principal components (PCs). First 9 PCs capturing 90.6% of variation in torso shape in the LIFE-Adult cohort, shown as the maximum positive (left) and negative (right) deviations from the sample mean. Blue and red regions represent areas that protrude less, or more than the average torso shape, respectively.

PCA produced 9 PCs that captured 90.6% of the total torso shape variation within the sample (Fig 3). Fig 3 shows the maximum deviations from the average torso shape of the sample along each of these PCs, with blue and red regions on the images representing areas that protrude less, or more than the average torso shape of the cohort, respectively. The torso shape features present within the sample resemble those identified in previous studies [30, 35, 41], such as variations in anterior-posterior weighting (PC1), abdominal roundness (PC3), lateral asymmetry of the torso due to variations in posture (PC4) and mass distributions along the length of the torso segment (PC2). The extremes of PC2 appear to represent differences between typical males and female torso shapes, and seems to correspond to the shape differences observed by Torres-Tamayo et al., with males shown to have wider upper torso than lower torso, while females demonstrate the opposite trend [39, 40]. Regression of the residuals of each shape PC on sex were not statistically significant for most PCs (PC1, 2, 4, 5, 8 and 9) (p < 0.001). While the regression of residuals within shape PCs 3, 6 and 7 on sex were shown to be statistically significant (p < 0.001) (S1 Table). Statistical difference testing found significant differences between males and females for all body measures and shape PCs analysed in this study (S1 Table). Finding such clear differences supported the decision to stratify all analyses by sex, therefore we calculated sex-specific means and standard deviations for all torso shape PC scores and body measures of participants (as performed in a previous study by Pavlova et al. [54]).

### Correlations between body measures and shape PCs

**Fig 4:**
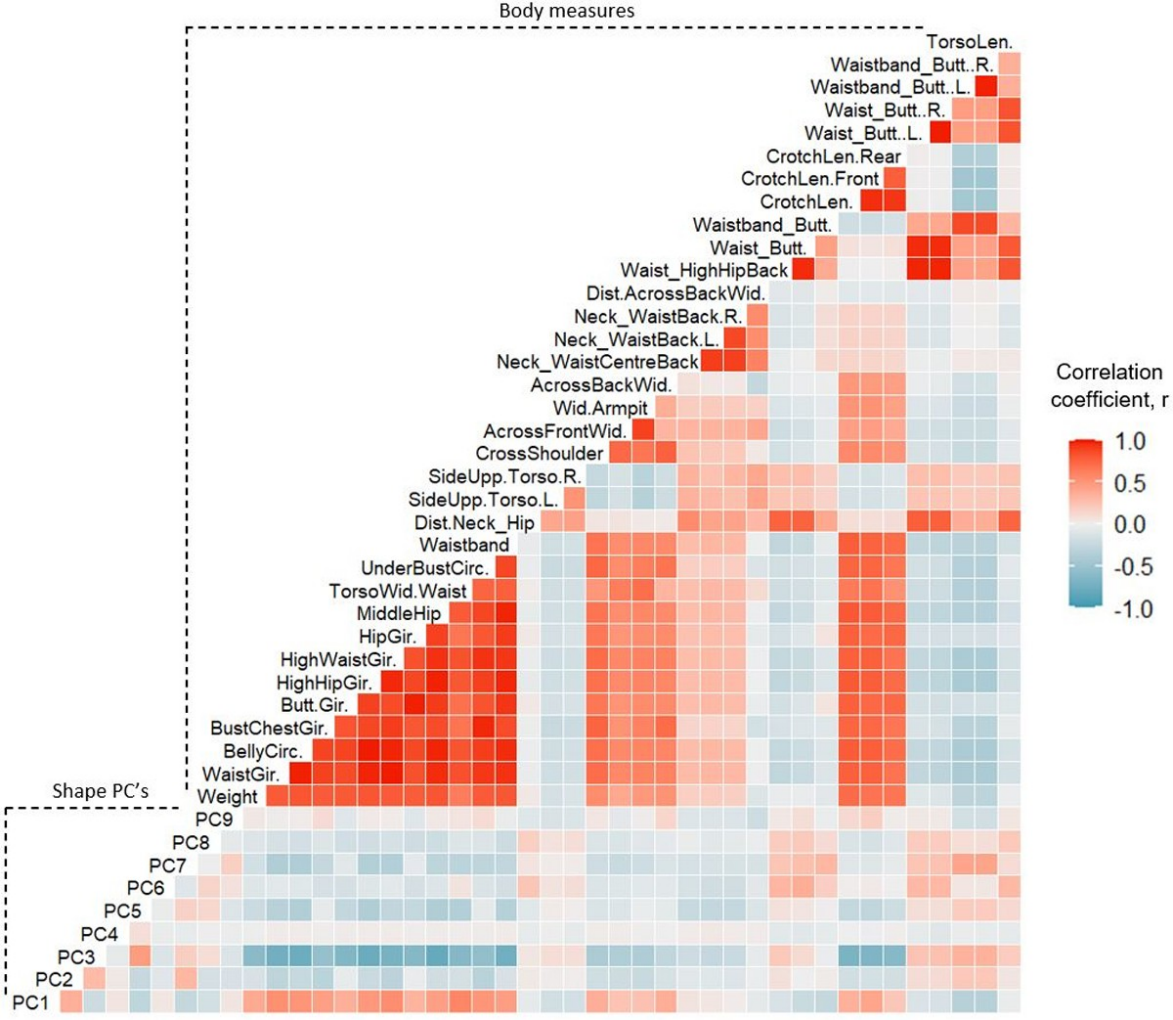
Correlation matrix of male participant measures. Strength of linear relationships between traditional body measures and shape PCs for male participants. Dark red panels indicate a strong positive correlation, dark blue panels indicate a strong negative correlation, lighter panels indicate weak correlation.

**Fig 5:**
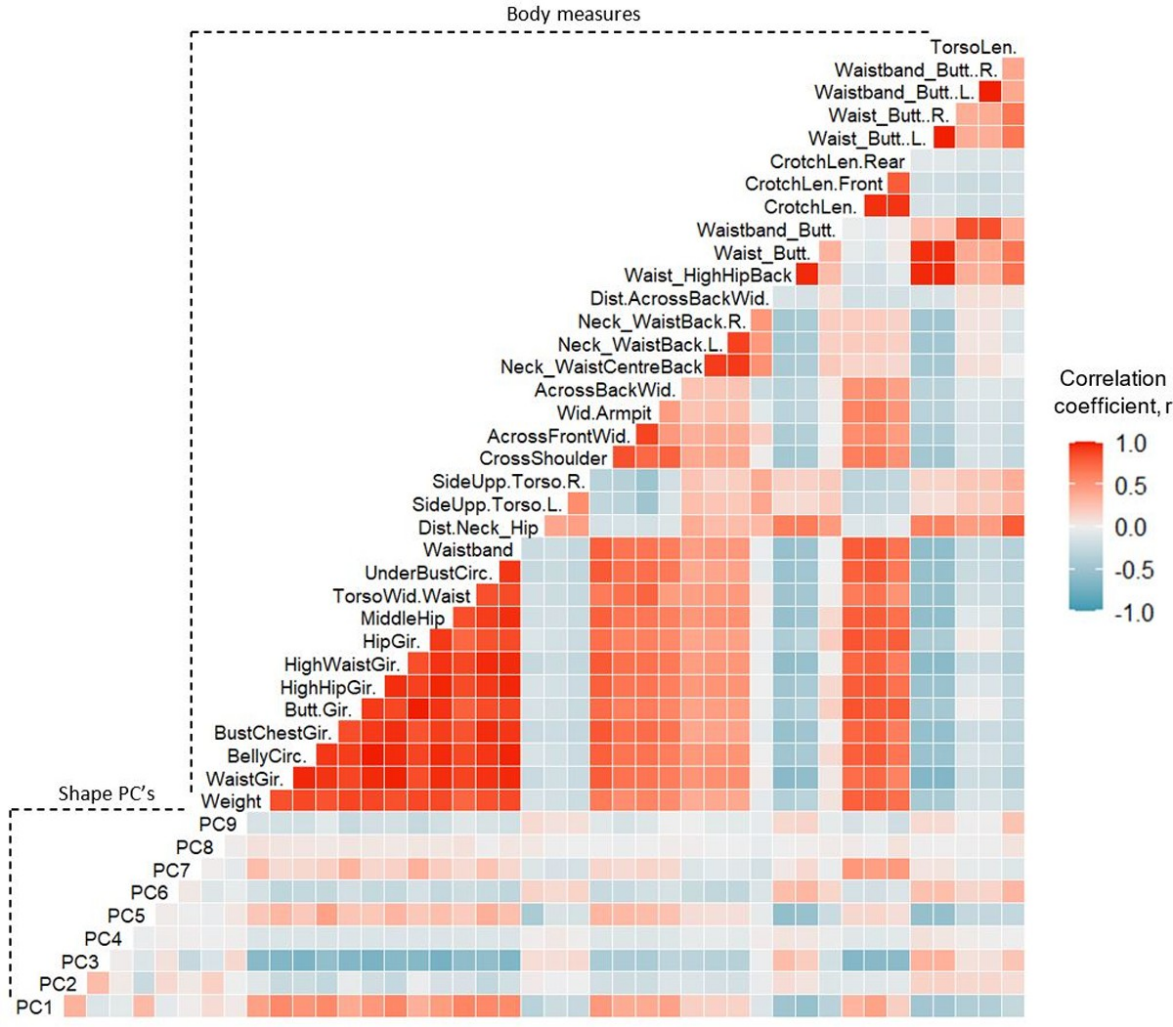
Correlation matrix of female participant measures. Strength of linear relationships between traditional body measures and shape PCs for female participants. Dark red panels indicate a strong positive correlation, dark blue panels indicate a strong negative correlation, lighter panels indicate weak correlation.

Pearson correlation coefficient (*r*) matrices of relationships between body measures, as well as relationships between body measures and shape PCs are provided as S1 Table and presented visually in Figs 4 and 5 for males and females, respectively. Girth measures taken along the torso segment demonstrated the highest correlations with each other and body weight, in both males (*r*: median = 0.88; IQR = 0.13) and females (*r*: median = 0.90; IQR = 0.09) (p < 0.001). While torso length and distance measures, such as side upper torso length and waist-buttock distance, showed the weakest correlations with other body measures, both for males (*r*: median = 0.17; IQR = 0.18) and females (*r*: median = 0.24; IQR = 0.28) (p < 0.001). Shape PC3 demonstrated the strongest correlations with body measures; in particular, with girth measures around the abdominal region, such as waist girth and middle hip girth, for males (*r*: median = 0.76; IQR = 0.06) and females (*r*: median = 0.67; IQR = 0.06) (p < 0.001). Shape PC’s 1, 2 and 5 demonstrated weak to moderate correlations with torso girth and width measures, males (*r*: median = 0.30; IQR = 0.20) and females (*r*: median = 0.27; IQR = 0.23) (p < 0.001). The remaining torso shape PCs demonstrated only weak linear correlations with body measures. Though the original PCs detected within the entire sample of participants were orthogonal and therefore uncorrelated due to the nature of PCA, some weak-moderate correlations were observed between the shape PCs for males and females separately. The PC scores of the male and female participants were recentred as separate z-scores, due to observed sex differences, the subsequent change in the correlational structure between individuals’ shape PCs caused some correlation to emerge between them. However, this change was not deemed to have affected the subsequent analysis, because the ordinal association between body measures and shape PCs were not affected.

### Partial least squares regression analysis

It was determined by estimating the mean squared prediction error by 10-fold cross-validation that six PLS components were required to maximise the prediction accuracy, R^2^, of the PLS regression models, while minimising the risk of overfitting for each shape PC. The developed PLS regression models demonstrated that traditional body measures were able to explain different amounts of variance within each of the torso shape PCs for male and female participants (S1 Table and Fig 9). Shape PC3 had the largest amount of variation explained by body measures, (R^2^ = 0.84) and (R^2^ = 0.73) for males and females, respectively. Conversely, variations in shape PC4, which represent tilting of the torso from side to side and are unlikely to be captured by body measures, were almost completely unexplained by body measures, for both males (R^2^ = 0.03) and females (R^2^ = 0.04).

**Fig 6:**
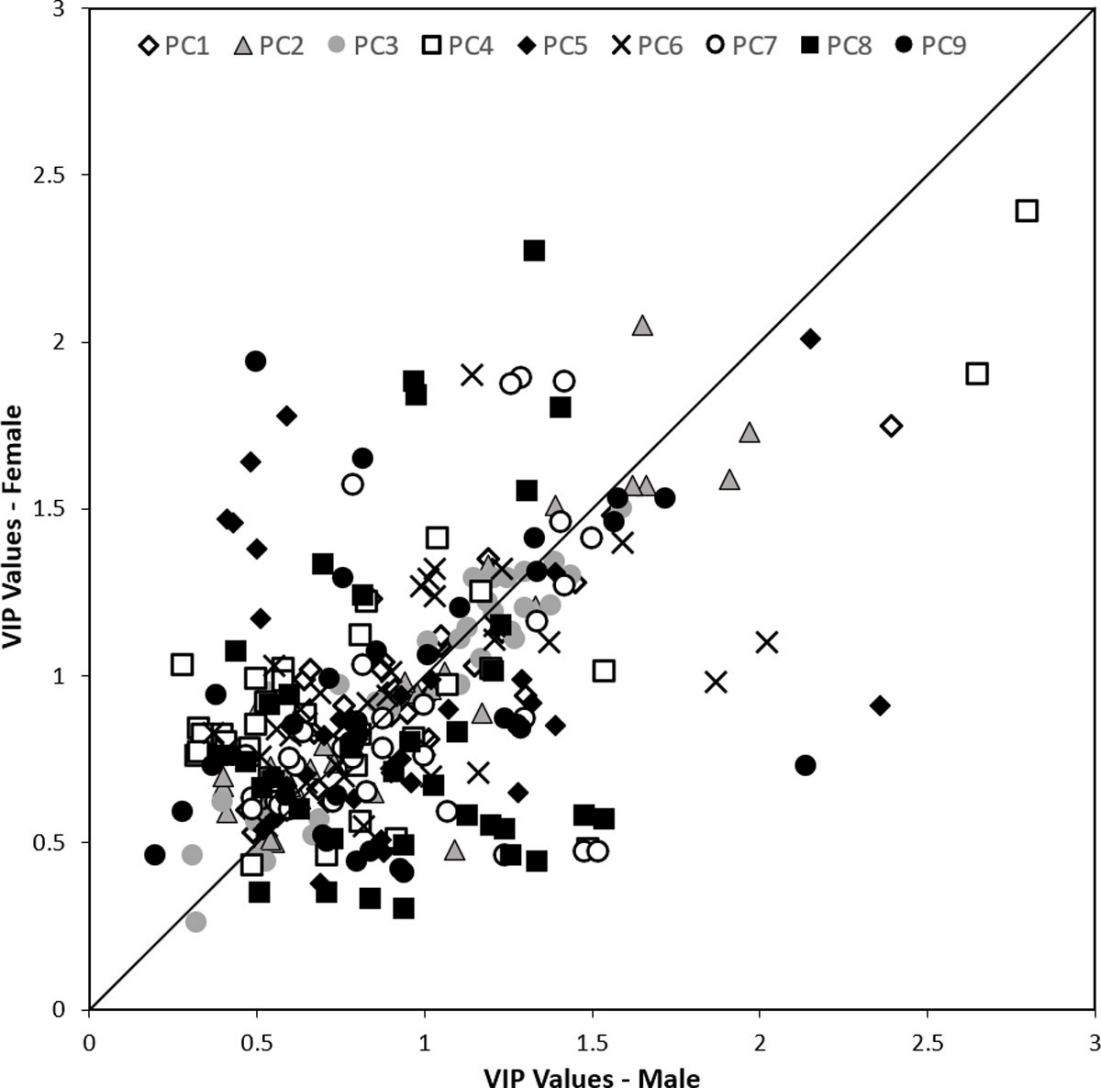
Variable importance in projection (VIP) of body measures within PLS regression models. Comparison of calculated VIP statistic values within the PLS regression model for each torso shape PC between male and females.

Within the male PLS regression models, between 44–65% of body measures demonstrated VIP values ≥ 0.8, while in the female PLS regression models between 41–82% of body measures had VIP values ≥ 0.8 (see S1 Table and Fig 6). Within the male and female PLS regression models, different combinations of body measures contributed more strongly to the prediction of shape PCs. This suggests that males and females demonstrate different relationships between traditional measures of their torso and shape characteristics, with different morphological variations caused by changes in traditional body measures. However, Fig 6 does show that VIP values appear to be in closer agreement between males and females for shape PCs 1–3. This suggests that traditional body measures contribute to the prediction of these components of torso shape similarly for both sexes. Whereas, for shape PCs 4–9 there were greater levels of disagreement in how body measures contribute to the prediction of shape between males and females, with these higher PCs possibly representing sexual dimorphisms in torso variation.

### Model between traditional anthropometrics and shape PCs

**Fig 7:**
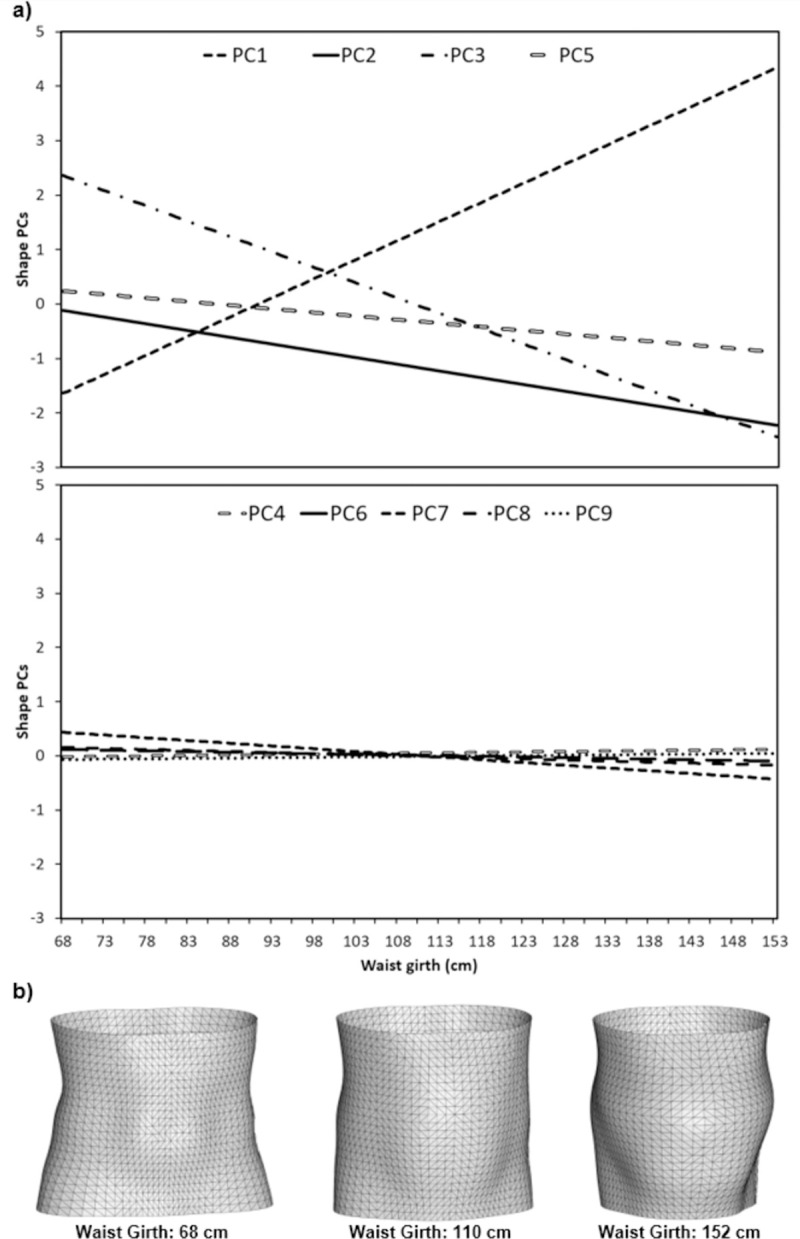
Relationship between body measures and shape PCs for male participants. (a) Predicted changes in shape PCs according to PLSR models. (b) Reconstructed torso shapes for males with waist girths of 68, 110 and 152 cm.

**Fig 8:**
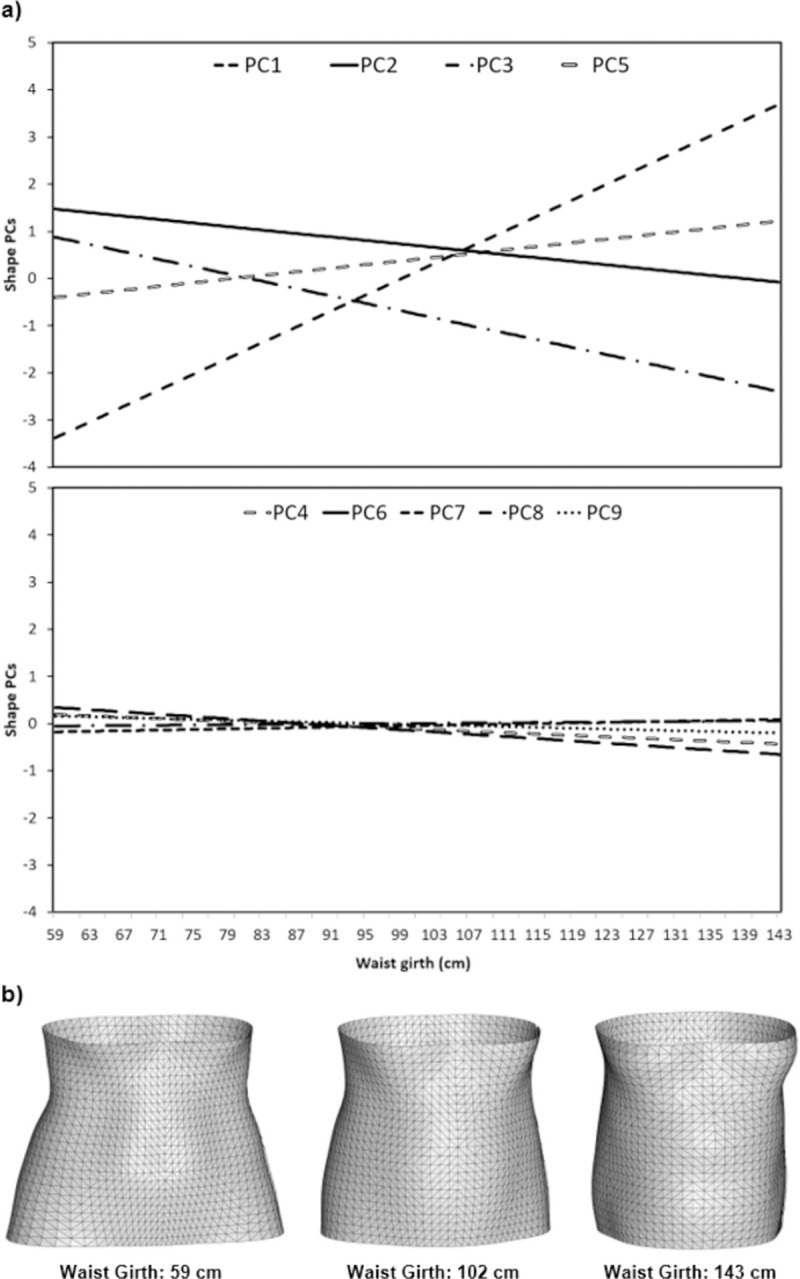
Relationship between body measures and shape PCs for female participants. (a) Predicted changes in shape PCs according to PLSR models. (b) Reconstructed torso shapes for females with waist girths of 59, 102 and 143 cm.

Figs 7A and 8A present the predicted variations torso shape PCs 1–9 that correspond to unit changes in waist girth measures for males and females, respectively. There are strong relationships between body measures and shape PC’s 1, 2, 3 and 5 with these features changing significantly with changes in traditional body measures, for both males and females. Conversely, shape PC’s 4, 6, 7, 8 and 9 demonstrated weak relationships with body measures. The predicted changes in torso shape PCs with changes in body measures are visualised as reconstructed torso meshes for the smallest, median and largest waist girth values within the participant sample for males (Fig 7B) and females (Fig 8B), respectively. Increases in waist girth correspond to observed increases in mass and curvature on the anterior aspect of the torso segment, for both males and females. However, males appear to exhibit these changes primarily in the central region of the torso, while females exhibit these changes initially in the lower torso and bust region. However, the largest female torsos demonstrate changes in shape also occur across the rest of the anterior aspect of the torso segment for female participants.

**Fig 9:**
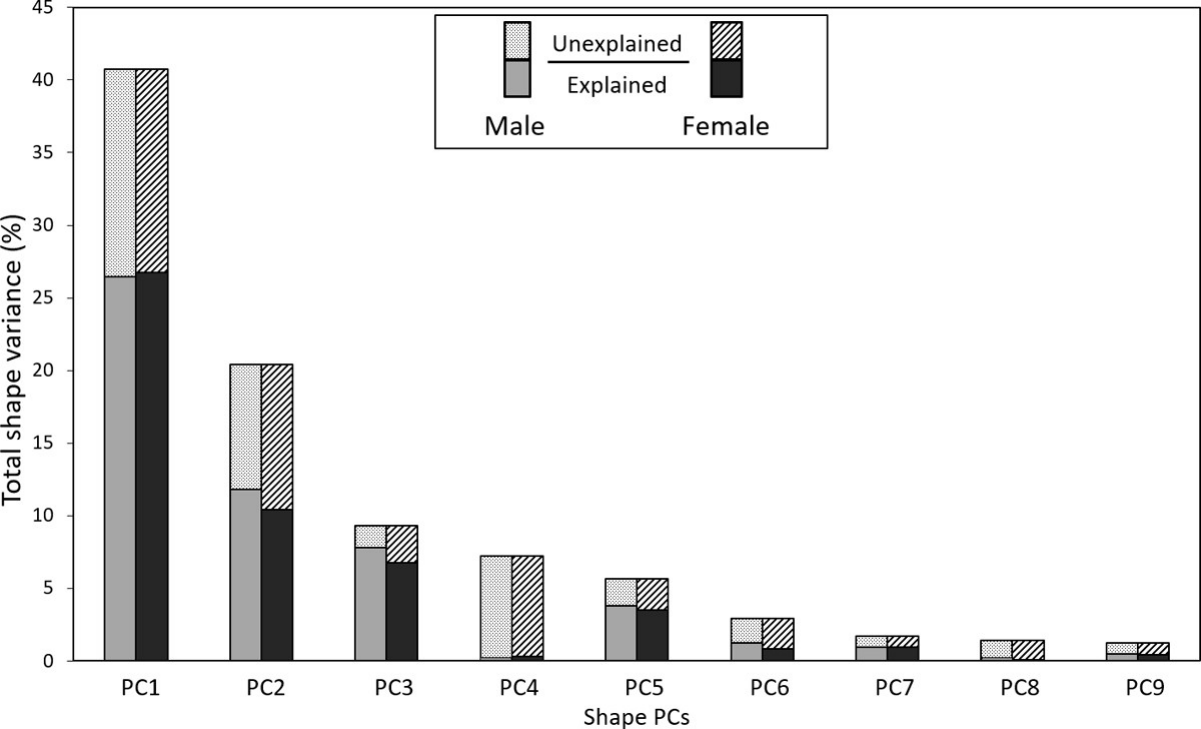
Proportions of total torso shape variation within LIFE dataset explained and unexplained by each torso shape PC for males and females.

As shown in Figs 7 and 8, the strongest relationships were observed between body measures and shape PCs 1, 2, 3 and 5, which collectively explain 76.1% of the total variability in torso shape for participants in the LIFE Adult dataset. Though large proportions of PCs 1, 2, 3 and 5 are explained by the PLSR model (Male: PC1 (65%), PC2 (58%), PC3 (84%), PC5 (67%); Female: PC1 (66%), PC2 (51%), PC3 (73%), PC5 (62%)) there are still considerable amounts of variance within each of these shape PCs which is unexplained (Fig 9). The torso shape variance that is unexplained within PCs 1, 2, 3 and 5, accounts for 26.2% and 28.7% of the total torso shape variability in the LIFE dataset for males and females, respectively. In comparison, the combined torso shape variance (both explained and unexplained) within PCs 4, 6, 7, 8 and 9 accounts for 14.5% of the total torso shape variability in the LIFE dataset. This suggests that although torso shape variation not associated with traditional body measures is more subtle, it still represents a considerable proportion of possible variation in torso shape, which could contain useful morphological information.

## Discussion

Variations in shape of the human torso cannot be wholly explained by variations in traditional body measures. This investigation demonstrated that 49.92% and 47.46% of the observed torso shape variation within the LIFE dataset were explained by changes in body measures for males and females, respectively. Linear combinations of body measures in the PLS regression models explained over 50% of the variation in each of shape PCs 1, 2, 3 and 5 for males (PC1 (65%), PC2 (58%), PC3 (84%), PC5 (67%)) and females (PC1 (66%), PC2 (51%), PC3 (73%), PC5 (62%)). The VIP values of body measures in the PLS regression models suggest that torso girth measures contributed most to the prediction of torso shape. This would suggest that traditional body measures are a good predictor of torso shape for approximately 50% large proportion of the population that follow the expected trend.

However, the results of this investigation also show that nearly 40% of the observed torso shape variation within the LIFE dataset could not be explained by changes in body measures. PCs 4 and 8, represented torso features which were almost completely unexplained by changes in body measures. From visualisations of these shape features, PC4 appears to represent lateral asymmetries of the torso. These variations could be the result of participants leaning to the left or right during the scanning procedure, a common issue within digital anthropometry, or could indicate issues such as scoliosis, which size measures would likely not capture. PC8 appears to represent subtle, localised variations in body shape on the inferior anterior aspect of the torso, which could relate to distributions of body fat or lean mass along the length of the torso that are not captured by current techniques. This would suggest that there are individuals within the population that exhibit torso shape characteristics which do not match the predicted trend according to existing body measures alone, with additional measures therefore required to evaluate other aspects of their morphology.

Shape PCs 1, 2, 3 and 5 had the strongest associations with body measures and represented over 76% of the total torso shape variability within the LIFE dataset. In comparison, the remaining PCs 4, 6, 7, 8 and 9 only accounted for 14.5% of the total torso shape variability in the LIFE dataset. Though large proportions of PCs 1, 2, 3 and 5 are explained by changes in body measures, the variance within these PCs that remains unexplained still accounts for over 26% of the total shape variability within the sample—which is greater than the variation within the other PCs combined. In addition, greater levels of agreement were observed between VIP values in the PLS regressions for first few PCs between males and females, while the higher order PCs showed less agreement. A possible reason for this could be that the first few PCs represent large-scale, high amplitude shape variations that vary in a consistent manner between males and females, while the higher order PCs represent more subtle, lower amplitude variations which characterise sexually dimorphic aspects of torso shape that differ in an unexplained manner between males and females. When assessing the relationships between unexplained variations in torso shape and sex, it was demonstrated that the unexplained variance within some of the torso shape PCs were more related to sexual dimorphism than others. For example, the regression of unexplained torso shape variations within PC2 on sex was not statistically significant (R^2^ = 0.001, p = 0.024), suggesting that variations within the unexplained component of PC2 were not due to body shape differences between males and females. PC2 appears to correspond to the observed thoraco-pelvic covariation observed by Torres-Tamayo et al., [40] who also found that sex differences were not significant within the unexplained component of this aspect of torso shape. On the other hand, the regression of unexplained shape variations within PCs 6 and 7 on sex were statistically significant (R^2^ = 0.002, p < 0.001) and (R^2^ = 0.004, p < 0.001), respectively. This may suggest that the unexplained torso shape variations within these PCs could be driven primarily by differences in torso shape between males and females, rather than other health-related factors. What is not yet known is whether the more subtle, largely unexplained shape PCs (6, 7, 8 and 9) are more significant in describing an individual’s health profile (for example) than the proportion of unexplained variance within the larger PCs (1, 2, 3 and 5).

The role of future research will be to determine which aspects of body shape are more important with regards to predictive power and may hold additional information that is of interest in the assessment of health risk.

Currently, practitioners conducting clinical and population-based health screenings rely upon traditional anthropometrics and derived indices, to estimate quantities of abdominal obesity and classify individuals according to their risk of cardio-metabolic health issues [4, 14]. Adverse metabolic effects, such as insulin resistance or dyslipidemia, are likely to result from both dysfunctional abdominal subcutaneous and visceral fat accumulation through the release and excess flux of fatty acids [4, 55, 56]. Though it is currently unknown what the unexplained variations in torso shape identified in this study represent in terms of physical health, the information they provide could identify subtle, localised features of body surface topography that are related to the shape of skeletal structures, body fat distributions and associated health risks. Further study is required to establish these relationships. Also, due to the random distribution of unexplained shape variations throughout the participant sample, individuals classed as having different levels of cardio-metabolic risk, according to traditional measures, could in fact exhibit the same torso shape characteristics. The ability to identify shape features that may occur in individuals of many different sizes may be key to identifying people with poor metabolic health that are missed by current anthropometric screening methods which rely primarily on traditional body measures alone. Consequently, measuring variations in body shape to a greater level of precision than existing tools could enable clinical practitioners to perform more individualised population-level diagnoses of cardio-metabolic health risk. For example, the identification of individuals classed as being normal weight but with abdominal obesity and poor metabolic health.

Though LIFE-Adult has one of the largest collections of 3D body imaging data currently available, comprising an extensive range of body types, a limitation of this dataset is its lack of ethnic diversity. By design, the aim of LIFE-Adult was an extensive phenotyping of urban individuals from Leipzig, a city in Eastern Germany, and therefore participants were primarily of Central European origin [2]. This is significant, since it has been established that components of body composition that are expressed as body shape, such as fat-lean mass ratio, fat distribution and associated health risk varies significantly across ethnic groups at a given BMI [11, 57]. In addition, only a small proportion of participants within the LIFE-Adult study were under the age of 40 (~4%) [2]. It has been shown that although male body shape remains relatively consistent within BMI classifications between early adulthood and old age, female body shape changes from more of an hourglass body shape in early adulthood, to having greater levels of abdominal fat deposition and central obesity as they become older, regardless of their BMI [58]. As such further study is required to assess individuals from a wider range of ages and ethnicities, to determine whether the findings of this investigation are representative of the wider population.

## Conclusions

The results of this investigation suggest that although large amounts of torso shape variation can be explained by changes in body measures, geometric morphometric methods can identify variations in human morphology, which cannot be explained by traditional body measures. It is posited that these unexplained variations in torso shape could be related to the quantities and distributions of excess abdominal fat mass, as well as associated health risks that aren’t identified by body measures currently used in practice. The aim of future work will be to further investigate the underlying causes of variations in human body shape and evaluate the benefits of improved body measurements in medical applications, such as obesity classification and epidemiology.

## Supporting information

S1 File Source code. MATLAB code to process raw 3D scan data and landmark files used to calculate torso shape PCs.
(PDF)

S1 Table List of body size measures. Information about body size measures included in the torso segment analysis; statistical difference testing between males and females for all size and shape anthropometrics; correlations between size and shape anthropometrics; as well as R^2^ values and variable importance in projection (VIP) statistic scores for the predictor size variables used within each PLSR model for males and females.
(XLSX)

S2 Table Processed data table. Contains participant characteristics, size measures and body shape principal component scores of all 9,209 participants.
(XLSX)

## References

[1] Stewart AD. Kinanthropometry and body composition: A natural home for three-dimensional photonic scanning. Journal of Sports Sciences. 2010;28(5):455–7. doi: 10.1080/02640411003661304 20419588

[2] Loeffler M, Engel C, Ahnert P, Alfermann D, Arelin K, Baber R, et al. The LIFE-Adult-Study: Objectives and design of a population-based cohort study with 10,000 deeply phenotyped adults in Germany. BMC Public Health. 2015;15(1):1–14. doi: 10.1186/s12889-015-1983-z 26197779 PMC4509697

[3] Treleaven P. Sizing us up. IEEE Spectrum. 2004;41(April):29–31.

[4] Piché ME, Poirier P, Lemieux I, Després JP. Overview of Epidemiology and Contribution of Obesity and Body Fat Distribution to Cardiovascular Disease: An Update. Progress in Cardiovascular Diseases. 2018;61(2):103–13. doi: 10.1016/j.pcad.2018.06.004 29964067

[5] Duren DL, Sherwood RJ, Czerwinski SA, Lee M, Choh AC, Siervogel RM, et al. Body composition methods: Comparisons and interpretation. Journal of Diabetes Science and Technology. 2008;2(6):1139–46. doi: 10.1177/193229680800200623 19885303 PMC2769821

[6] Hume PA, Ackland T. Physical and Clinical Assessment of Nutritional Status. In: Nutrition in the Prevention and Treatment of Disease. Elsevier; 2017. p. 71–84.

[7] Albrizio A. Biometry and anthropometry: from Galton to constitutional medicine. Journal of Anthropological Sciences. 2007;85:101–23.

[8] Quetelet A. Anthropom{é}trie ou Mesure des diff{é}rentes facult{é}s de l’homme. C. Muquardt; 1870.

[9] World Health Organisation. Obesity: preventing and managing the global epidemic Report of a WHO Consultation (WHO Technical Report Series 894). 2000. 11234459

[10] World Health Organisation (WHO). WHO | Waist Circumference and Waist–Hip Ratio. Report of a WHO Expert Consultation. Geneva, 8–11 December 2008. 2008(December):8–11.

[11] Wells JCK. Ethnic variability in adiposity, thrifty phenotypes and cardiometabolic risk: addressing the full range of ethnicity, including those of mixed ethnicity. Obesity reviews. 2012 Dec;13(S2):14–29. doi: 10.1111/j.1467-789X.2012.01034.x 23107256

[12] Kissebah AH, Vydelingum N, Murray R, Evans DJ, Kalkhoff RK, Adams PW. Relation of Body Fat Distribution to Metabolic Complications of Obesity. The Journal of Clinical Endocrinology & Metabolism. 1982 February;54(2):254–60. doi: 10.1210/jcem-54-2-254 7033275

[13] Neeland IJ, Poirier P, Després JP. Cardiovascular and Metabolic Heterogeneity of Obesity: Clinical Challenges and Implications for Management. Circulation. 2018;137(13):1391–406. doi: 10.1161/CIRCULATIONAHA.117.029617 29581366 PMC5875734

[14] Swainson MG, Batterham AM, Tsakirides C, Rutherford ZH, Hind K. Prediction of whole-body fat percentage and visceral adipose tissue mass from five anthropometric variables. PLoS ONE. 2017;12(5):1–12. doi: 10.1371/journal.pone.0177175 28493988 PMC5426673

[15] Jensen SM, Mølgaard C, Ejlerskov KT, Christensen LB, Michaelsen KF, Briend A. Validity of anthropometric measurements to assess body composition, including muscle mass, in 3-year-old children from the SKOT cohort. Maternal and child nutrition. 2015 Jul;11(3):398–408. doi: 10.1111/mcn.12013 23167700 PMC6860213

[16] Jaeschke L, Steinbrecher A, Pischon T. Measurement of waist and hip circumference with a body surface scanner: Feasibility, validity, reliability, and correlations with markers of the metabolic syndrome. PLoS ONE. 2015;10(3):1–16. 10.1371/journal.pone.0119430 PMC4352076 25749283

[17] Wells JCK, Treleaven P, Cole TJ. BMI compared with 3-dimensional body shape: The UK National Sizing Survey. American Journal of Clinical Nutrition. 2007;85(2):419–25. doi: 10.1093/ajcn/85.2.419 17284738

[18] Rosenzweig JL, Bakris GL, Berglund LF, Hivert M, Horton ES, Kalyani RR, et al. Primary Prevention of ASCVD and T2DM in Patients at Metabolic Risk: An Endocrine Society* Clinical Practice Guideline. The Journal of Clinical Endocrinology & Metabolism. 2019;104(9):3939–85. 10.1210/jc.2019-01338 31365087

[19] Dumas A, Costigan PA, Wicke J. Trunk density profile estimates from dual X-ray absorptiometry. 2008;41:861–7. doi: 10.1016/j.jbiomech.2007.10.022 18082166

[20] Wicke J, Dumas GA. Influence of the Volume and Density Functions Within Geometric Models for Estimating Trunk Inertial Parameters. 2010:26–31. doi: 10.1123/jab.26.1.26 20147755

[21] Nevill AM, Duncan MJ, Lahart IM, Sandercock GR. Scaling waist girth for differences in body size reveals a new improved index associated with cardiometabolic risk. Scandinavian Journal of Medicine and Science in Sports. 2017;27(11):1470–6. doi: 10.1111/sms.12780 27726187

[22] Coutinho T, Goel K, Corrêa De Sá D, Carter RE, Hodge DO, Kragelund C, et al. Combining body mass index with measures of central obesity in the assessment of mortality in subjects with coronary disease: Role of "normal weight central obesity". Journal of the American College of Cardiology. 2013;61(5):553–60. doi: 10.1016/j.jacc.2012.10.035 23369419

[23] Goh LGH, Dhaliwal SS, Welborn TA, Lee AH, Della PR. Anthropometric measurements of general and central obesity and the prediction of cardiovascular disease risk in women: A cross-sectional study. BMJ Open. 2014;4(2). doi: 10.1136/bmjopen-2013-004138 24503301 PMC3918987

[24] Nevill AM, Stewart AD, Olds T, Duncan MJ. A new waist-to-height ratio predicts abdominal adiposity in adults. Research in Sports Medicine. 2020;28(1):15–26. doi: 10.1080/15438627.2018.1502183 30044132

[25] Löffler-Wirth H, Willscher E, Ahnert P, Wirkner K, Engel C, Loeffler M, et al. Novel anthropometry based on 3D-bodyscans applied to a large population based cohort. PLoS ONE. 2016;11(7):1–20. doi: 10.1371/journal.pone.0159887 27467550 PMC4965021

[26] Soileau L, Bautista D, Johnson C, Gao C, Zhang K, Li X, et al. Automated anthropometric phenotyping with novel Kinect-based three-dimensional imaging method: Comparison with a reference laser imaging system. European Journal of Clinical Nutrition. 2016;70(4):475–81. doi: 10.1038/ejcn.2015.132 26373966

[27] Tsang B, Chan CK, Taylor G, Tsangc B, Taylor KC. Kinanthropometry study of the physique of disciplined personnel. International Journal of Clothing Science and Technology. 2000;12(2):144–60.

[28] Wells JCK, Ruto A, Treleaven P. Whole-body three-dimensional photonic scanning: A new technique for obesity research and clinical practice. International Journal of Obesity. 2008;32(2):232–8. doi: 10.1038/sj.ijo.0803727 17923860

[29] Price GM, Uauy R, Breeze E, Bulpitt CJ, Fletcher AE. Weight, shape, and mortality risk in older persons: elevated waist-hip ratio, not high body mass index, is associated with a greater risk of death. The American Journal of Clinical Nutrition. 2006 August;84(2):449–60. doi: 10.1093/ajcn/84.1.449 16895897

[30] Ng BK, Sommer MJ, Wong MC, Pagano I, Nie Y, Fan B, et al. Detailed 3-dimensional body shape features predict body composition, blood metabolites, and functional strength: the Shape Up! studies. The American Journal of Clinical Nutrition. 2019:1–11. 10.1093/ajcn/nqz218 PMC6885475 31553429

[31] Daanen HAM, Ter Haar FB. 3D whole body scanners revisited. Displays. 2013;34(4):270–5.

[32] Hamad M, Thomassey S, Bruniaux P. A new sizing system based on 3D shape descriptor for morphology clustering. Computers and Industrial Engineering. 2017;113:683–92.

[33] Gupta D. Anthropometry and the design and production of apparel: an overview. In: Anthropometry, Apparel Sizing and Design. Elsevier; 2014. p. 34–66.

[34] Pleuss JD, Talty K, Morse S, Kuiper P, Scioletti M, Heymsfield SB, et al. A machine learning approach relating 3D body scans to body composition in humans. European Journal of Clinical Nutrition. 2019;73(2):200–8. doi: 10.1038/s41430-018-0337-1 30315314 PMC8108117

[35] Ruto A, Lee M, Buxton B. Comparing principal and independent modes of variation in 3D human torso shape using PCA and ICA. ICA Research Network. 2006:3–6.

[36] Douros I. Calculating the Curvature Shape Characteristics of the Human Body from 3D Scanner Data [dissertation].; 2004.

[37] Lu Y, Mcquade S, Hahn JK. 3D Shape-based Body Composition Prediction Model Using Machine Learning.; 2018. 10.1109/EMBC.2018.8513261 PMC6538417 30441235

[38] Adams D, Rohlf JL, Slice D. A field comes of age: geometric morphometrics in the 21 st century. Hystrix, the Italian Journal of Mammalogy. 2013;24(1):7–14.

[39] Torres-Tamayo N, Garcia-Martinez D, Nalla S, Barash A, Williams S, Blanco-Perez E, et al. The torso integration hypothesis revisited in Homo sapiens: Contributions to the understanding of hominin body shape evolution. American Journal of Physical Anthropology. 2018;167:777–90. doi: 10.1002/ajpa.23705 30259957

[40] Torres-Tamayo N, Martelli S, Schlager S, Garcia-Martinez D, Sanchis-Gimeno JA, Mata-Escolano F, et al. Assessing thoraco-pelvic covariation in Homo sapiens and Pantroglodytes: A 3D geometric morphometric approach. American Journal of Biological Anthropology. 2020;173:514–34. 10.1002/ajpa.24103 32864759

[41] Thelwell M, Chiu CY, Bullas A, Hart J, Wheat J, Choppin S. How shape - based anthropometry can complement traditional anthropometric techniques: a cross - sectional study. Scientific Reports. 2020:1–11. doi: 10.1038/s41598-019-56847-4 32699270 PMC7376175

[42] von Elm E, Altman DG, Egger M, Pocock SJ, Gøtzsche PC, Vandenbroucke JP. The Strengthening the Reporting of Observational Studies in Epidemiology (STROBE) statement: guidelines for reporting observational studies. Journal of Clinical Epidemiology. 2008 April;61(4):344–9. doi: 10.1016/j.jclinepi.2007.11.008 18313558

[43] Office IS, inventor; ISO 20685–1:2018(en) - 3-D scanning methodologies for internationally compatible anthropometric databases—Part 1: Evaluation protocol for body dimensions extracted from 3-D body scans. 2018.

[44] Kuehnapfel A, Ahnert P, Loeffler M, Broda A, Scholz M. Reliability of 3D laser-based anthropometry and comparison with classical anthropometry. Scientific Reports. 2016;6(May):1–11. doi: 10.1038/srep26672 27225483 PMC4880916

[45] Stewart AD, Marfell-Jones M, Olds T, E Al. International standards for anthropometric assessment. Lower Hutt, New Zealand: International Society for the Advancement of Kinanthropometry. 2011:125f.

[46] Choppin S, Clarkson S, Bullas A, Thelwell M, Heller B, Wheat J. Anatomical and principal axes are not aligned in the torso: Considerations for users of geometric modelling methods. Journal of Biomechanics. 2020;114:110151. doi: 10.1016/j.jbiomech.2020.110151 33307355

[47] Thelwell M, Bullas A, Kuehnapfel A, Hart J, Ahnert P, Wheat J, et al. Allometry Between Measures of Body Size and Shape in a Large Population-Based Cohort. Ascona, Switzerland: Hometrica Consulting—Dr. Nicola D’Apuzzo; November 2020.

[48] Clarkson S, Wheat J, Heller B, Choppin S. Assessing the suitability of the Microsoft Kinect for calculating person specific body segment parameters Assessing the Suitability of the Microsoft Kinect for Calculating Person Specific Body Segment Parameters.; 2014.

[49] de Boor C. A Practical Guide to Splines (Applied Mathematical Sciences). Springer-Verlag New York; 1978.

[50] Sullivan GM, Feinn R. Using Effect Size—or Why the P Value Is Not Enough. Journal of Graduate Medical Education. 2012 September;4(3):279–82. doi: 10.4300/JGME-D-12-00156.1 23997866 PMC3444174

[51] Wold S, Ruhe A, Wold H, Dunn I, J W. The Collinearity Problem in Linear Regression. The Partial Least Squares (PLS) Approach to Generalized Inverses. SIAM Journal on Scientific and Statistical Computing. 1984 September;5(3):735–43.

[52] Wold S, Sjöström M, Eriksson L. PLS-regression: A basic tool of chemometrics. Chemometrics and Intelligent Laboratory Systems. 2001;58(2):109–30.

[53] Farrés M, Platikanov S, Tsakovski S, Tauler R. Comparison of the variable importance in projection (VIP) and of the selectivity ratio (SR) methods for variable selection and interpretation. Journal of Chemometrics. 2015;29(10):528–36.

[54] Pavlova AV, Saunders FR, Muthuri SG, Gregory JS, Barr RJ, Martin KR, et al. Statistical shape modelling of hip and lumbar spine morphology and their relationship in the MRC National Survey of Health and Development. Journal of Anatomy. 2017;231(2):248–59. doi: 10.1111/joa.12631 28561274 PMC5522893

[55] Dulloo AG, Jacquet J, Solinas G, Montani J, Schutz Y. Body composition phenotypes in pathways to obesity and the metabolic syndrome. International Journal of Obesity. 2010 Dec;34(S2):S4–S17. doi: 10.1038/ijo.2010.234 21151146

[56] Després JP. Body fat distribution and risk of cardiovascular disease: An update. Circulation. 2012;126(10):1301–13. doi: 10.1161/CIRCULATIONAHA.111.067264 22949540

[57] Wells JCK. The Metabolic Ghetto: An Evolutionary Perspective on Nutrition, Power Relations and Chronic Disease. Cambridge University Press; 2016.

[58] Wells JCK, Cole TJ, Treleaven P. Age-variability in Body Shape Associated With Excess Weight: The UK National Sizing Survey. Obesity (Silver Spring, Md.). 2008 Feb;16(2):435–41. doi: 10.1038/oby.2007.62 18239656

