# Supplementary material for: Modelling of human torso shape variation inferred by geometric morphometrics
Source: PLoS One. 2022 Mar 10;17(3):e0265255. doi: 10.1371/journal.pone.0265255 (PMC8912174; doi:10.1371/journal.pone.0265255)
Supplement: S1 File — MATLAB code to process raw 3D scan data and landmark files used to calculate torso shape PCs. (PDF) [file pone.0265255.s001.pdf]

TorsoShapeProcessingCode.m

```
%% STAGE 1 - INITIAL CONVERSION OF FILES FROM .OBJ TO .PLY %%
%% convert .obj to .ply
filelist=dir('*.obj');

n = numel(filelist);
meshlabserverpath='"H:\MeshLab/meshlabserver.exe"';
for i =1:n
    objfilename=filelist(i).name;
    plyfilename=strrep(objfilename,'.obj','.ply');
    disp([meshlabserverpath,' -i ',objfilename ,' -o ',plyfilename])
    system([meshlabserverpath,' -i ',objfilename ,' -o ',plyfilename])
end

%% STAGE 2 - MATCH SCAN FILES WITH EXPORTED LANDMARKS AND INITIAL PROCESSING %%
%% read in useful data from measurement excel files
filelist=dir(['Initial files' '/*.ply']); % wherever scan data files were stored
NumOverall = numel(filelist);
ix=strfind(filelist(1).name,'.');
for i = 1:NumOverall
    name=filelist(i).name(1:ix-1);
    PartNums{i,1} = name;
end

%% read in feature point .xml files and organise cordinate data
filelist_features=dir(['Initial files' '/*.xml']); % wherever raw landmark data files 
were stored
for i = 1:NumOverall
    name=filelist_features(i).name;
    features = xml2struct(name);
    filelist_features(i).landmarksdatastruct = features;
    m = numel(filelist_features(i).landmarksdatastruct.landmarks.scan.landmark);
    for p = 1:m
        filelist_features(i).features{p,1} = 
filelist_features(i).landmarksdatastruct.landmarks.scan.landmark{1,p}.Attributes.name;
        filelist_features(i).features{p,2} = 
filelist_features(i).landmarksdatastruct.landmarks.scan.landmark{1,p}.Attributes.x;
        filelist_features(i).features{p,2} = 
str2double(filelist_features(i).features{p,2});
        filelist_features(i).features{p,3} = 
filelist_features(i).landmarksdatastruct.landmarks.scan.landmark{1,p}.Attributes.y;
        filelist_features(i).features{p,3} = 
str2double(filelist_features(i).features{p,3});
        filelist_features(i).features{p,4} = 
filelist_features(i).landmarksdatastruct.landmarks.scan.landmark{1,p}.Attributes.z;
        filelist_features(i).features{p,4} = 
str2double(filelist_features(i).features{p,4});
        filelist_features(i).features{p,5} = [filelist_features(i).features{p,2} 
filelist_features(i).features{p,3} filelist_features(i).features{p,4}];
    end
    filelist(i).featurepoints = filelist_features(i).features;
end

%% rotate scan files and segment torso
tic
for i = 1:NumOverall
    Torso = pcread(filelist(i).name);
    R = roty(90);
    m = numel(filelist(i).featurepoints(:,1));
    Torso = Torso.Location*R;
    filelist(i).featurepoints{1,5} = filelist(i).featurepoints{1,5}*R;
    for q = 2:m
```

```
    filelist(i).featurepoints{q,5} = filelist(i).featurepoints{q,5}*R;
end

%% Orientate 3D scan
%ID shoulder points on body scan
LeftShoulderID = 'shoulder L';
for q = 1:m
    featurename=filelist(i).featurepoints{q,1};
    tf = strcmp(featurename,LeftShoulderID);
    TrueFalse_feature(q,1) = tf;
end
index = find(TrueFalse_feature(:,1) == 1);
filelist(i).LeftShoulder = filelist(i).featurepoints{index,5};

RightShoulderID = 'shoulder R';
for q = 1:m
    featurename=filelist(i).featurepoints{q,1};
    tf = strcmp(featurename,RightShoulderID);
    TrueFalse_feature(q,1) = tf;
end
index = find(TrueFalse_feature(:,1) == 1);
filelist(i).RightShoulder = filelist(i).featurepoints{index,5};

% align torso according to shoulder coordinates
A = filelist(i).LeftShoulder;
B = filelist(i).RightShoulder;
v3 = B-A;
unitv3 = v3/norm(v3);
ay = atan2(norm(cross(unitv3,[1,0,0])),dot(unitv3,[1,0,0]));
thetay = rad2deg(ay);
if A(2)>B(2)
    thetay = thetay*-1;
end
Rz = rotz(thetay);
filelist(i).TorsoTwist = thetay;
Torso = Torso*Rz;
filelist(i).featurepoints{1,5} = filelist(i).featurepoints{1,5}*Rz;
for q = 2:m
    filelist(i).featurepoints{q,5} = filelist(i).featurepoints{q,5}*Rz;
end
% ID neck front point on body scan
NeckID = 'neck front point';
for q = 1:m
    featurename=filelist(i).featurepoints{q,1};
    tf = strcmp(featurename,NeckID);
    TrueFalse_feature(q,1) = tf;
end
index = find(TrueFalse_feature(:,1) == 1,1,'first');
filelist(i).Neck = filelist(i).featurepoints{index,5};
NeckHeight = filelist(i).Neck;

% ID buttock height
ButtockID = '11330';
for q = 1:m
    featurename=filelist(i).featurepoints{q,1};
    tf = strcmp(featurename,ButtockID);
    TrueFalse_feature(q,1) = tf;
end
index = find(TrueFalse_feature(:,1) == 1);
filelist(i).Buttock = filelist(i).featurepoints{index,5};
ButtockHeight = filelist(i).Buttock;
% ID nipples
RightNippleID = 'nippel R';
```

```
    for q = 1:m
        featurename=filelist(i).featurepoints{q,1};
        tf = strcmp(featurename,RightNippleID);
        TrueFalse_feature(q,1) = tf;
    end
    index = find(TrueFalse_feature(:,1) == 1);
    TF2 = isempty(index);
    if TF2 ==1
        filelist(i).NippleRight = [0.1 nan nan];
    else
        filelist(i).NippleRight = filelist(i).featurepoints{index,5};
    end
    NippleRight = filelist(i).NippleRight;
    LeftNippleID = 'nippel L';
    for q = 1:m
        featurename=filelist(i).featurepoints{q,1};
        tf = strcmp(featurename,LeftNippleID);
        TrueFalse_feature(q,1) = tf;
    end
    index = find(TrueFalse_feature(:,1) == 1);
    TF2 = isempty(index);
    if TF2 ==1
        filelist(i).NippleLeft = [-0.1 nan nan];
    else
        filelist(i).NippleLeft = filelist(i).featurepoints{index,5};
    end
    NippleLeft = filelist(i).NippleLeft;
    % calculate proportionate torso length and find central anterior & posterior
    % points to define midpoint of axis set
    [Torso,BodyLength,AxisBodyLength,Spine,Midpoint,SegmentBodyLength,Rz] =
MidpointAxisTorso(Torso,NeckHeight,ButtockHeight,NippleRight,NippleLeft);
    filelist(i).BodyLength = BodyLength;
    filelist(i).AxisBodyLength = AxisBodyLength;
    filelist(i).SegmentBodyLength = SegmentBodyLength;
    filelist(i).featurepoints{1,5} = filelist(i).featurepoints{1,5} - Midpoint;
    for q = 2:m
        filelist(i).featurepoints{q,5} = filelist(i).featurepoints{q,5} - Midpoint;
    end
    filelist(i).featurepoints{1,5} = filelist(i).featurepoints{1,5}*Rz;
    for q = 2:m
        filelist(i).featurepoints{q,5} = filelist(i).featurepoints{q,5}*Rz;
    end
    Midpoint = Midpoint - Midpoint;

    % ID Waist height on body scan
    LeftWaistID = 'waist girth left';
    for q = 1:m
        featurename=filelist(i).featurepoints{q,1};
        tf = strcmp(featurename,LeftWaistID);
        TrueFalse_feature(q,1) = tf;
    end
    index = find(TrueFalse_feature(:,1) == 1);
    filelist(i).LeftWaist = filelist(i).featurepoints{index,5};

    TF = isempty(filelist(i).LeftWaist);
    if TF == 1
        RightWaistID = 'waist girth right';
    for q = 1:m
        featurename=filelist(i).featurepoints{q,1};
        tf = strcmp(featurename,RightWaistID);
        TrueFalse_feature(q,1) = tf;
    end
    index = find(TrueFalse_feature(:,1) == 1);
```

```matlab
    filelist(i).RightWaist = filelist(i).featurepoints{index,5};
    Bottom = filelist(i).RightWaist(3);
    else
        Bottom = filelist(i).LeftWaist(3);
    end

% change vertical position of torso according to height of waist girth markers
    Origin = [0, 0, Bottom];
    Torso = Torso - Origin.*ones(length(Torso),3);
    filelist(i).LeftWaist = filelist(i).LeftWaist - Origin;
    filelist(i).Spine = Spine - Origin;
    filelist(i).Midpoint = Midpoint - Origin;
    filelist(i).featurepoints{1,5} = filelist(i).featurepoints{1,5} - Origin;
    for q = 2:m
        filelist(i).featurepoints{q,5} = filelist(i).featurepoints{q,5} - Origin;
    end

%% segment body scan

    % ID buttock height
    ButtockID = '11330';
    for q = 1:m
        featurename=filelist(i).featurepoints{q,1};
        tf = strcmp(featurename,ButtockID);
        TrueFalse_feature(q,1) = tf;
    end
    index = find(TrueFalse_feature(:,1) == 1);
    filelist(i).Buttock = filelist(i).featurepoints{index,5};
    ButtockHeight = filelist(i).Buttock;

    % ID neck front point on body scan
    NeckID = 'neck front point';
    for q = 1:m
        featurename=filelist(i).featurepoints{q,1};
        tf = strcmp(featurename,NeckID);
        TrueFalse_feature(q,1) = tf;
    end
    index = find(TrueFalse_feature(:,1) == 1,1,'first');
    filelist(i).Neck = filelist(i).featurepoints{index,5};
    NeckHeight = filelist(i).Neck;

    % ID shoulder
    LeftShoulderID = 'shoulder L';
    for q = 1:m
        featurename=filelist(i).featurepoints{q,1};
        tf = strcmp(featurename,LeftShoulderID);
        TrueFalse_feature(q,1) = tf;
    end
    index = find(TrueFalse_feature(:,1) == 1);
    filelist(i).LeftShoulder = filelist(i).featurepoints{index,5};
    LeftShoulderOffset = filelist(i).LeftShoulder(2);

    RightShoulderID = 'shoulder R';
    for q = 1:m
        featurename=filelist(i).featurepoints{q,1};
        tf = strcmp(featurename,RightShoulderID);
        TrueFalse_feature(q,1) = tf;
    end
    index = find(TrueFalse_feature(:,1) == 1);
    filelist(i).RightShoulder = filelist(i).featurepoints{index,5};
    RightShoulderOffset = filelist(i).RightShoulder(2);
    Shoulders = [LeftShoulderOffset;RightShoulderOffset];
    ShoulderPoint = mean(Shoulders);
```

```
    % ID elbows
    LeftElbowID = 'elbow L';
    for q = 1:m
        featurename=filelist(i).featurepoints{q,1};
        tf = strcmp(featurename,LeftElbowID);
        TrueFalse_feature(q,1) = tf;
    end
    index = find(TrueFalse_feature(:,1) == 1);
    TF2 = isempty(index);
    if TF2 ==1
        filelist(i).LeftElbow = [nan nan nan];
    else
        filelist(i).LeftElbow = filelist(i).featurepoints{index,5};
    end
    LeftElbowOffset = filelist(i).LeftElbow(2);

    RightElbowID = 'elbow R';
    for q = 1:m
        featurename=filelist(i).featurepoints{q,1};
        tf = strcmp(featurename,RightElbowID);
        TrueFalse_feature(q,1) = tf;
    end
    index = find(TrueFalse_feature(:,1) == 1);
    TF2 = isempty(index);
    if TF2 ==1
        filelist(i).RightElbow = [nan nan nan];
    else
        filelist(i).RightElbow = filelist(i).featurepoints{index,5};
    end
    RightElbowOffset = filelist(i).RightElbow(2);
    Elbows = [LeftElbowOffset;RightElbowOffset];
    ElbowPoint = mean(Elbows);

    % perform segmentation procedure
    [Torso,LengthTorso,Origin] =
SegmentTrim_LIFE(Torso,ButtockHeight,NeckHeight,ShoulderPoint,ElbowPoint,SegmentBodyLengt
h);
    filelist(i).LeftShoulder = filelist(i).LeftShoulder - Origin;
    filelist(i).RightShoulder = filelist(i).RightShoulder - Origin;
    filelist(i).Spine = filelist(i).Spine - Origin;
    filelist(i).Midpoint = filelist(i).Midpoint - Origin;
    filelist(i).LengthTorso = LengthTorso;
    filelist(i).TrimmedBodyScan = Torso;

end
toc
%% STAGE 3 - SCALING ALL TRIMMED TORSO POINTCLOUDS TO UNIFORM SIZE AND EXTRACTING DATA
SLICES %%
% determine smallest participant in sample for scaling
NumOverall = numel(filelist);
TorsoLengths = zeros(NumOverall,1);
for i = 1:NumOverall
   TorsoLengths(i,1) =  filelist(i).LengthTorso;
end
SmallestTorso = min(TorsoLengths(:));
NumSlices = 25;
SliceStep = SmallestTorso/(NumSlices-1);

%%
tic
for i = 1:NumOverall
%% Extract data slices & resample
```

```
Torso = filelist(i).TrimmedBodyScan;
LengthTorso = filelist(i).LengthTorso;
Interval = abs(LengthTorso/(NumSlices-1));
[Slices,ALL3D] = SliceExtractTorso(Torso,Interval,NumSlices,SliceStep);
filelist(i).SLICES = Slices;
filelist(i).ALL3D = ALL3D;

%% Convert landmark coordinates into polar coordinates
for z = 1:NumSlices
    slice = filelist(i).SLICES{z};
    [theta, rho] = cart2pol(slice(:,1),slice(:,2));
    filelist(i).POLARSLICE{z} = [theta rho];
end

%% Perform Fourier analysis
for slice = 1:NumSlices
        theta = filelist(i).POLARSLICE{slice}(:,1);
        [theta,I] = sort(theta);
        rho = filelist(i).POLARSLICE{slice}(:,2);
        rho = rho(I);
        [Y,P,Y_trim] = FourierAnalysis(rho,theta);
        filelist(i).FFT_full{slice} = Y;
        filelist(i).FFT_power{slice} = P;
        filelist(i).FFT_trim{slice} = Y_trim;
end
%Flatten the trimmed fft coefficients for the next step
filelist(i).FFT_trim_ALL = cell2mat(filelist(i).FFT_trim);

end
toc
%% STAGE 4 - SEPARATE USEFUL PROCESSED DATA INTO DATA STRUCTURES
% create overall filelist with only information required for further processing
for i = 1:NumOverall
    BothGenderFilelist(i).Name = filelist(i).name(1:ix-1);
    BothGenderFilelist(i).BMI = filelist(i).BMI;
    BothGenderFilelist(i).WHR = filelist(i).WHR;
    BothGenderFilelist(i).Gender = filelist(i).Gender;
    BothGenderFilelist(i).Age = filelist(i).Age;
    BothGenderFilelist(i).ALL3D = filelist(i).ALL3D;
    BothGenderFilelist(i).FFT_trim_ALL = filelist(i).FFT_trim_ALL;
end

%% STAGE 5 - DIMENSION REDUCTION AND PCA TO EXTRACT SHAPE FEATURES
% Create matrices of Fourier Coefficients for male and female participants

[FourierVar{1:NumOverall}] = BothGenderFilelist(:).FFT_trim_ALL;
FourierVar = cell2mat(FourierVar');
imag_part = imag(FourierVar);
real_part = real(FourierVar);

%% Perform PCA
% create matrix with real and imaginary values of fourier coefficients

Fourier_Real_Imag = zeros(1,length(FourierVar(1,:))*2);
for i = 1:NumOverall
    Fourier_Real_Imag(i,1:2:end-1) = real_part(i,:);
    Fourier_Real_Imag(i,2:2:end) = imag_part(i,:);
    BothGenderFilelist(i).Fourier_Real_Imag = Fourier_Real_Imag(i,:);
end

% Calculating Coefficients of PC and their respected variances
[coeff, score, latent, tsquared, explained, mu] =
pca(BothGender_FourierStruct(1).Real_Imag);
```

```
BothGender_FourierStruct(1).AllPCAScores = score;
BothGender_FourierStruct(1).Coeff = coeff;
BothGender_FourierStruct(1).Latent = latent;
BothGender_FourierStruct(1).Tsquared = tsquared;
BothGender_FourierStruct(1).ExplainedVariance = explained;
BothGender_FourierStruct(1).Mu = mu;

%% Add PCA shape features into data structures
for i = 1:NumOverall
    BothGenderFilelist(i).Fourier_PCA = BothGender_FourierStruct(1).AllPCAScores(i,:);
end
```

MidpointAxisTorso.m

```
function [Torso,BodyLength,AxisBodyLength,Spine,Midpoint,SegmentBodyLength,Rz] =
MidpointAxisTorso(Torso,NeckHeight,ButtockHeight,NippleRight,NippleLeft)

   % calculate distance from body top to buttock
    BodyLength = NeckHeight(3)-ButtockHeight(3);
    % calculate 70% of body length to find height to create coordinate axis
    AxisBodyLength = BodyLength*0.7;
    % calculate 60% of body length to find height for segmentation
    SegmentBodyLength = BodyLength*0.6;

    % take slice to find front and rear points on torso
    sliceheight = ButtockHeight(3)+ AxisBodyLength;
    sli = getslice(Torso,sliceheight,[0 0 0],3,0.005);

    % check to see if nipples are in the right place
    nipplediff = norm(NippleRight(3)-NippleLeft(3));
    if nipplediff > 0.05
        NippleRight = [0.1 0.1 sliceheight-0.1];
        NippleLeft = [-0.1 0.1 sliceheight-0.1];
    end

    % find central point on rear of torso
    reartorso = min(sli(:,2));
    index = find(sli(:,2) < (reartorso+0.1));
    reartorso = sli(index,:);
    v3 = NippleLeft-NippleRight;
    Mid = v3/2;
    MidNipple = NippleRight+Mid;
    LeftMarker = MidNipple(1)-0.025;
    RightMarker = MidNipple(1)+0.025;
    index = find(reartorso(:,1) > (NippleLeft(1)+0.065));
    rearright = reartorso(index,:);
    index = find(rearright(:,1) < (NippleRight(1)-0.065));
    rearcentre = rearright(index,:);
    Spine = max(rearcentre(:,2));
    index = find(rearcentre(:,2) == Spine,1,'first');
    Spine = rearcentre(index,:);

    % find central point on front of torso at mid chest level
    fronttorso = max(sli(:,2));
    index = find(sli(:,2) > (fronttorso-0.1));
    fronttorso = sli(index,:);
    index = find(fronttorso(:,1) > LeftMarker);
    frontright = fronttorso(index,:);
    index = find(frontright(:,1) < RightMarker);
    frontcentre = frontright(index,:);
    number = numel(frontcentre(:,1));
    for i = 1:number
        Dist = pdist([Spine;frontcentre(i,:)]);
        Vectors(i) = Dist;
    end
    ShortestDist = min(Vectors(:));
    index = find(Vectors == ShortestDist,1,'first');
    Sternum = frontcentre(index,:);
    Spine(3) = Sternum(3);

    % find vector from spine to sternum
    SpinetoSternumVect = Spine-Sternum;
    Mid = SpinetoSternumVect/2;
    Midpoint = Sternum+Mid;
    SpinetoMidpointVect = Midpoint-Spine;
```

```
    % extract slice at xiphoid process level
    sliceheight = ButtockHeight(3)+ SegmentBodyLength;
    sli = getslice(Torso,sliceheight,[0 0 0],3,0.005);

    % find spine point on rear of torso and new midpoint at xiphoid level
    reartorso = min(sli(:,2));
    index = find(sli(:,2) < (reartorso+0.1));
    reartorso = sli(index,:);
    index = find(reartorso(:,1) > LeftMarker);
    rearright = reartorso(index,:);
    index = find(rearright(:,1) < RightMarker);
    rearcentre = rearright(index,:);
    Spine = max(rearcentre(:,2));
    index = find(rearcentre(:,2) == Spine,1,'first');
    Spine = rearcentre(index,:);
    Spine(3) = sliceheight;
    Midpoint = Spine-SpinetoMidpointVect;

    % centre torso according to midpoint of anterior and posterior markers
    Torso = Torso - Midpoint.*ones(length(Torso),3);
    Spine = Spine - Midpoint;
    unitv3 = SpinetoMidpointVect/norm(SpinetoMidpointVect);
    ay = atan2(norm(cross(unitv3,[0,1,0])),dot(unitv3,[0,1,0]));
    thetay = rad2deg(ay);
    thetay = double(thetay);
    Rz = rotz(thetay);
    Spine = Spine*Rz;

end
```

SegmentTrimTorso.m

```
function [Torso,LengthTorso,Origin] = SegmentTrimTorso(x,y,z,n,o,q)

% Trim point cloud vertically to just show torso for analysis
a = find(x(:,3)<y(3));     % remove all points where x is less than buttock height
x(a,:)= [];

a = find(x(:,3)>z(3));     % remove all points where x is greater than neck height
x(a,:) = [];

% determine 60% torso height for segmentation
bodylength60 = y(3)+q;

% determine the width of arms to remove for initial processing
indmax = max(x(:,1));      % finds most right lateral point in scan which should be on the
arm
indmin = min(x(:,1));      % finds most left lateral point in scan which should be on the
arm

index = find(x(:,3)>=0);
UpperTorso = x(index,:); %determines the upper torso above the waist
index = find(x(:,3)<0);
LowerTorso = x(index,:); %determines the lower torso below the waist
R = rotx(90);
Rotated = UpperTorso*R;              % rotates torso to enable location of armpit
shoulderoffset = n;
Lower = [0, 0, shoulderoffset];
Rotated = Rotated + Lower.*ones(length(Rotated),3); % moves torso so that the slice is
taken through the line of the shoulders
maxheight = max(Rotated(:,2));
heightfactor = maxheight-0.05;
index = find(Rotated(:,2)> heightfactor);   % removes uppermost part of the scan to help
find the armpit
Rotated(index,:) = [];
elbowoffset = o;
diff = shoulderoffset-elbowoffset;  % determines the offset between shoulders and elbows
absdiff = abs(diff);
if absdiff > 0.1
    slice = getslice(Rotated,0.02,[0 0 0],3,0.005);
else
    slice = getslice(Rotated,0,[0 0 0],3,0.004);   % gets slice of upper torso to get
frontal profile
end
sli = slice;
armwidth = indmax-indmin;       % determine how much of the lateral points to remove from
rotated scan
armwidth50 = armwidth*0.55;
widthfactor = armwidth*0.25;
factor = indmax-widthfactor;        % remove most lateral points to remove as much of the
arm as possible before next stage
index = find(sli(:,1)>factor);
sli(index,:) = [];
factor = indmin+widthfactor;        % remove most lateral points to remove as much of the
arm as possible before next stage

index = find(sli(:,1)<factor);
sli(index,:) = [];              % removes parts of slice which don't relate to the sides
of torso

epsilon = 0.03;
minpts = 10;
```

```
IDX = dbscan(sli,epsilon,minpts);  % finds connected components of slice that we want to
keep to find armpits
UniqueVals = unique(IDX);
misc = find(UniqueVals == -1);
UniqueVals(misc) = [];
DiffComponents = numel(UniqueVals);
% check to see if there are different parts
if DiffComponents == 1
    widthfactor = 0.1;
    factor = indmax-widthfactor;
    index = find(slice(:,1)>factor);
    slice(index,:) = [];
    factor = indmin+widthfactor;
    index = find(slice(:,1)<factor);
    slice(index,:) = [];
    %redo dbscan
    epsilon = 0.03;
    minpts = 10;
    IDX = dbscan(slice,epsilon,minpts);  % finds connected components of slice that we
want to keep to find armpits
    UniqueVals = unique(IDX);
    misc = find(UniqueVals == -1);
    UniqueVals(misc) = [];
    DiffComponents = numel(UniqueVals);
    for i = 1:DiffComponents
        index = find(IDX == i);
        NumComp(i) = numel(index);
    end
    sortedComps = sort(NumComp,'descend');  % sort components from largest to smallest
    TorsoSide1 = sortedComps(1);            % define largest as one side of torso
    TorsoSide2 = sortedComps(2);            % define second largest as the other side of
torso
    index = find(NumComp == TorsoSide1,1,'first');
    TorsoSideComp1 = UniqueVals(index);
    if TorsoSide1 == TorsoSide2
        index = find(NumComp == TorsoSide2,1,'last');
    else
        index = find(NumComp == TorsoSide2);
    end
    TorsoSideComp2 = UniqueVals(index);
    index1 = find(IDX == TorsoSideComp1);
    index2 = find(IDX == TorsoSideComp2);
    TorsoSides = [slice(index1,:);slice(index2,:)];  % define the parts of slice which
are sides of torso
else
    for i = 1:DiffComponents
        index = find(IDX == i);
        NumComp(i) = numel(index);
    end
    sortedComps = sort(NumComp,'descend');  % sort components from largest to smallest
    TorsoSide1 = sortedComps(1);            % define largest as one side of torso
    TorsoSide2 = sortedComps(2);            % define second largest as the other side of
torso
    index = find(NumComp == TorsoSide1,1,'first');
    TorsoSideComp1 = UniqueVals(index);
    if TorsoSide1 == TorsoSide2
        index = find(NumComp == TorsoSide2,1,'last');
    else
        index = find(NumComp == TorsoSide2);
    end
    TorsoSideComp2 = UniqueVals(index);
    index1 = find(IDX == TorsoSideComp1);
    index2 = find(IDX == TorsoSideComp2);
```

```
    TorsoSides = [sli(index1,:);sli(index2,:)];  % define the parts of slice which are
sides of torso
end

index = find(TorsoSides(:,1) > 0);
right = [TorsoSides(index,1) TorsoSides(index,2)]; %determine which components are the
left and right sides of the torso
index = find(TorsoSides(:,1) < 0);
left = [TorsoSides(index,1) TorsoSides(index,2)];

% find the upper and lower points of the armpits to enable the arm segments to be removed
consistently

maxleft = max(left(:,2));           % find the max point on the left side which we can
take to be the top of the armpit
index = find(left(:,2) == maxleft,1,'first');
leftarmpitwidth = left(index,1);
index = find(UpperTorso(:,1) < 0);
LeftTorso = UpperTorso(index,:);
dist = 0.005;
for i = 0:40
    armpitslice = getslice(LeftTorso,(maxleft-(dist+(i*0.005))),[0 0 0],3,0.005);
    epsilon = 0.02;
    minpts = 10;
    IDX = dbscan(armpitslice,epsilon,minpts);  % finds connected components of slice that
we want to keep to find armpits
    UniqueVals = unique(IDX);
    misc = find(UniqueVals == -1);
    UniqueVals(misc) = [];
    DiffComponents = numel(UniqueVals);
    if DiffComponents > 1
        break
    end
end
leftarmpitbottom = maxleft-(dist+0.02+(i*0.005));
index = find(UpperTorso(:,1) == leftarmpitwidth);
leftarmpit = UpperTorso(index,:);
a = find(UpperTorso(:,1)<(leftarmpit(1)+0.012));          % remove all points above and
to the side of the armpit point
lateralleft = [UpperTorso(a,:) a];
b = find(lateralleft(:,3)>(leftarmpitbottom));
upperlateralleft = lateralleft(b,:);
unwantedrows = upperlateralleft(:,4);
UpperTorso(unwantedrows,:) = [];

maxright = max(right(:,2));  % find the max point on the right side which we can take to
be the top of the armpit
index = find(right(:,2) == maxright,1,'first');
rightarmpitwidth = right(index,1);
index = find(UpperTorso(:,1) > 0);
RightTorso = UpperTorso(index,:);
dist = 0.005;
for i = 0:40
    armpitslice = getslice(RightTorso,(maxright-(dist+(i*0.005))),[0 0 0],3,0.005);
    epsilon = 0.02;
    minpts = 10;
    IDX = dbscan(armpitslice,epsilon,minpts);  % finds connected components of slice that
we want to keep to find armpits
    UniqueVals = unique(IDX);
    misc = find(UniqueVals == -1);
    UniqueVals(misc) = [];
    DiffComponents = numel(UniqueVals);
    if DiffComponents > 1
```

```
        break
    end
end
rightarmpitbottom = maxright-(dist+0.02+(i*0.005));
index = find(UpperTorso(:,1) == rightarmpitwidth);
rightarmpit = UpperTorso(index,:);
a = find(UpperTorso(:,1)>(rightarmpit(1)-0.012));          % remove all points above and
to the side of the armpit point
lateralright = [UpperTorso(a,:) a];
b = find(lateralright(:,3)>(rightarmpitbottom));
upperlateralright = lateralright(b,:);
unwantedrows = upperlateralright(:,4);
UpperTorso(unwantedrows,:) = [];

x = [UpperTorso;LowerTorso];

% perform dbscan procedure of finding connected components
epsilon = 0.005;
minpts = 5;
IDX = dbscan(x,epsilon,minpts);
UniqueVals = unique(IDX);
misc = find(UniqueVals == -1);
UniqueVals(misc) = [];
DiffComponents = numel(UniqueVals);
for i = 1:DiffComponents
    index = find(IDX == i);
    NumComp(i) = numel(index);
end
LargestComp = max(NumComp);
index = find(NumComp == LargestComp);
torsocomp = UniqueVals(index);
a = find(IDX~=torsocomp);
x(a,:) = [];

% Remove points above scapula to assess performance
a = find(x(:,3)>bodylength60);    % remove all points where x is greater than 60% torso
height
x(a,:) = [];

% reposition torso so that buttock height is now at zero for slice
% extraction
Bottom = y(3);
Origin = [0, 0, Bottom];
x = x - Origin.*ones(length(x),3);
Top = max(x(:,3));
LengthTorso = Top;
Torso = x;

end
```

SliceExtractTorso.m

```
function [Slices,ALL3D] = SliceExtractTorso(x,y,w,v)

for n = 0:(w-1)
    sli = getslice(x,n*y);
    numpoints = numel(sli(:,1));
    if numpoints <= 100
        sli = getslice(x,n*y,[0 0 0],3,0.008);
        sli = double(sli);
    else
        sli = double(sli);
    end

    % split slice into left and right parts
    index = find(sli(:,1) > 0);
    sliright = sli(index,:);
    index = find(sli(:,1) < 0);
    slileft = sli(index,:);

    % determine if right side of slice is one part or if there is a gap
    % cause by arm join
    epsilon = 0.05;
    minpts = 10;
    IDX = dbscan(sliright,epsilon,minpts);
    UniqueVals = unique(IDX);
    misc = find(UniqueVals == -1);
    UniqueVals(misc) = [];
    DiffComponents = numel(UniqueVals);
    if  DiffComponents > 1
        avgy = mean(sliright(:,2));
        index = find(sliright(:,2)>avgy);
        front = sliright(index,:);
        index = find(sliright(:,2)<(avgy-0.03));
        rear = sliright(index,:);
        frontright = max(front(:,1));
        index = find(front(:,1) == frontright);
        FrontRight = front(index,:);
        rearright = max(rear(:,1));
        index = find(rear(:,1) == rearright);
        RearRight = rear(index,:);
        x_points = [FrontRight(1) rightmidx RearRight(1)];
        y_points = [FrontRight(2) rightmidy RearRight(2)];
        z_points = [FrontRight(3) rightmidz RearRight(3)];
        % interpolate using parametric splines
        pt = interparc(20,x_points,y_points,z_points,'spline');
        pt = double(pt);
        sliright = [sliright(:,:);pt];
    end

    epsilon = 0.05;
    minpts = 10;
    IDX = dbscan(slileft,epsilon,minpts);
    UniqueVals = unique(IDX);
    misc = find(UniqueVals == -1);
    UniqueVals(misc) = [];
    DiffComponents = numel(UniqueVals);
    if  DiffComponents > 1
        avgy = mean(slileft(:,2));
        index = find(slileft(:,2)>avgy);
        front = slileft(index,:);
        index = find(slileft(:,2)<(avgy-0.03));
        rear = slileft(index,:);
```

```
        frontleft = min(front(:,1));
        index = find(front(:,1) == frontleft);
        FrontLeft = front(index,:);
        rearleft = min(rear(:,1));
        index = find(rear(:,1) == rearleft);
        RearLeft = rear(index,:);
        x_points = [FrontLeft(1) leftmidx RearLeft(1)];
        y_points = [FrontLeft(2) leftmidy RearLeft(2)];
        z_points = [FrontLeft(3) leftmidz RearLeft(3)];
        % % interpolate using parametric splines
        pt1 = interparc(20,x_points,y_points,z_points,'spline');
        pt1 = double(pt1);
        slileft = [slileft(:,:);pt1];
    end

    sli = [sliright;slileft];
    circum = circumthigh(sli);
    slices{n+1,1}(:,1:2) = [circum(1,:).' circum(2,:).'];

    % find the most right and left mid points of extracted slices to be
    % used in slice interpolation if needed
    rightmax = max(sli(:,1));
    index = find(sli(:,1) == rightmax,1,'first');
    rightmidy = sli(index,2);
    rightmidz = sli(index,3);
    rightmidx = rightmax+0.005;

    leftmax = min(sli(:,1));
    index = find(sli(:,1) == leftmax,1,'first');
    leftmidy = sli(index,2);
    leftmidz = sli(index,3);
    leftmidx = leftmax-0.005;
end
%% Resample slices

    for z = 1:w

    Xx = slices{z,1}(:,1);
    Xy = slices{z,1}(:,2);
    px = Xx;
    py = Xy;
    % % interpolate using parametric splines
    pt = interparc(71,px,py,'spline');
    slicesresampled{z,1}(:,1) = pt(:,1);
    slicesresampled{z,1}(:,2) = pt(:,2);

    end

    for z = 1:w
    slicesresampled{z,1}(71,:) = [];
    end

%% Scale Shapes to uniform size
    for z = 1:w
    DistsquaredX = slicesresampled{z,1}(:,1:2).^2;
    CentroidSize(z,1) = sqrt(sum(DistsquaredX(:)));
    end

    SumCentroidSize = sum(CentroidSize);
    B = SumCentroidSize./w;

    for z = 1:w
        SLICES{z,1}(:,1:2) = slicesresampled{z,1}(:,1:2)./norm(B);
```

```
    end

    for slice = 1:w
        SLICEXY = SLICES{slice,1}(:,1:2);
        SLICE3D{slice} = [SLICEXY repmat(v*(slice-1),70,1)];
    end
    Slices = SLICE3D;
    ALL3D = cell2mat(SLICE3D');

end
```

FourierAnalysis.m

```
function [Y,P,Y_trim,f,reordered_rho] = FourierAnalysis(x,y)

        first_rho = x(1); end_rho = x(end);
        min_theta = min(y); max_theta = max(y);
        x = [end_rho; x; first_rho];
        y = [max_theta-2*pi(); y; min_theta+2*pi()];
        points_required = 70;
        % resample to the number of points required
        theta_resamp = linspace(-pi(),+pi(),points_required);
        rho_resamp = spline(y,x,theta_resamp);

        Fs = points_required;            % Sampling frequency

        L = points_required;      % Signal length

        N = points_required;
        Y = fft(rho_resamp);
        P = abs(Y/N);
        f = Fs*(0:(N/2))/N;
        Y_trim = Y(1:10);
        reordered_rho = rho_resamp';

end
```

getslice.m

```
function slice = getslice(points,distance,landmark,axis,thickness)
% slice = getslice(points,distance)
% This function returns a 3D point cloud slice along the y-axis by a
% distance 'd'
% Thickness is option, default value is 2 mm
if nargin < 5
    thickness = 0.002;
end

% Default axis is x
if nargin < 3
    axis = 3;
end

% landmark provides an 'origin' from which to calculate distance, if it's
% not included it's set as (0,0,0)
if nargin < 3
    landmark = [0,0,0];
end


inslice = points(:,axis) > landmark(axis) +(distance - thickness/2) &
points(:,axis) < landmark(axis) +(distance + thickness/2);

slice = points(inslice,:);
```

circumthigh.m

```
function [slice_smooth,circumf,points_ctrd] = circumthigh(slice)

% Find centroid of points
CTR = centroid(slice);
% Transform points with centroid as origin
slice = [slice(:,1) - CTR(1) slice(:,2) - CTR(2)];



% Convert slice to radial co-ordinates
[THETA, RHO] = cart2pol(slice(:,1),slice(:,2));

% Sort the data to progress circularly
[THETA,I] = sort(THETA);
RHO = RHO(I);


% smooth the data with a smoothing spline
pp = csaps(THETA,RHO,0.999);

% Interpolate the data between 0 and 2pi()
THETA_SMTH = -pi():0.1:pi();
RHO_SMTH = fnval(pp,THETA_SMTH);

% Add the first data point to the end to complete the circle
THETA_SMTH = [THETA_SMTH THETA_SMTH(1)];

RHO_SMTH = [RHO_SMTH RHO_SMTH(1)];

%Transform back into cartesian
[X2,Y2] = pol2cart(THETA_SMTH,RHO_SMTH);


[circumf,~] = arclength(X2,Y2,'spline');

%CONVERT POINTS BACK INTO ORIGINAL AXES SET
slice_smooth = [X2 + CTR(1); Y2 + CTR(2)];
points_ctrd=[X2;Y2]';
```

centroid.m

```
function center = centroid(varargin)
%CENTROID Compute centroid (center of mass) of a set of points
%
%   PTS = centroid(POINTS)
%   PTS = centroid(PTX, PTY)
%   Computes the ND-dimensional centroid of a set of points.
%   POINTS is an array with as many rows as the number of points, and as
%   many columns as the number of dimensions.
%   PTX and PTY are two column vectors containing coordinates of the
%   2-dimensional points.
%   The result PTS is a row vector with Nd columns.
%
%   PTS = centroid(POINTS, MASS)
%   PTS = centroid(PTX, PTY, MASS)
%   Computes center of mass of POINTS, weighted by coefficient MASS.
%   POINTS is a Np-by-Nd array, MASS is Np-by-1 array, and PTX and PTY are
%   also both Np-by-1 arrays.
%
%   Example:
%   pts = [2 2;6 1;6 5;2 4];
%   centroid(pts)
%   ans =
%        4     3
%
%   See Also:
%   points2d, polygonCentroid
%
% ---------
% Author: David Legland
% e-mail: david.legland@grignon.inra.fr
% created the 07/04/2003.
% Copyright 2010 INRA - Cepia Software Platform.
%

%   HISTORY
%   2009-06-22 support for 3D points
%   2010-04-12 fix bug in weighted centroid
%   2010-12-06 update doc

%% extract input arguments

% use empty mass by default
mass = [];

if nargin==1
    % give only array of points
    pts = varargin{1};

elseif nargin==2
    % either POINTS+MASS or PX+PY
    var = varargin{1};
    if size(var, 2)>1
        % arguments are POINTS, and MASS
        pts = var;
        mass = varargin{2};
```

```
    else
        % arguments are PX and PY
        pts = [var varargin{2}];
    end

elseif nargin==3
    % arguments are PX, PY, and MASS
    pts = [varargin{1} varargin{2}];
    mass = varargin{3};
end

%% compute centroid

if isempty(mass)
    % no weight
    center = mean(pts);

else
    % format mass to have sum equal to 1, and column format
    mass = mass(:)/sum(mass(:));

    % compute weighted centroid
    center = sum(bsxfun(@times, pts, mass), 1);
    % equivalent to:
    % center = sum(pts .* mass(:, ones(1, size(pts, 2))));
End
```

Existing MATLAB codes

xml2struct.m

Wouter Falkena (2021). xml2struct (https://www.mathworks.com/matlabcentral/fileexchange/28518-xml2struct), MATLAB Central File Exchange. Retrieved December 11, 2021.

interparc.m

John D'Errico (2021). interparc (https://www.mathworks.com/matlabcentral/fileexchange/34874-interparc), MATLAB Central File Exchange. Retrieved December 11, 2021.

arclength.m

John D'Errico (2021). arclength (https://www.mathworks.com/matlabcentral/fileexchange/34871-arclength), MATLAB Central File Exchange. Retrieved December 11, 2021.
